# Supplementary material for: Evolutionary highways to persistent bacterial infection
Source: Nat Commun. 2019 Feb 7;10:629. doi: 10.1038/s41467-019-08504-7 (PMC6367392; doi:10.1038/s41467-019-08504-7)
Supplement: Supplementary file 1 — Supplementary Information [file 41467_2019_8504_MOESM1_ESM.pdf]

Supplementary Information
'Evolutionary highways to persistent bacterial infection'
by Bartell et al. (2019)

Supplementary Figures 1 - 7	pp 1 - 8
Supplementary Note 1 - Archetype Analysis	pp 9 - 21
Supplementary Note 2 - Generalized Additive Mixed Models	pp 22 - 38
Supplementary References	pp 39

Supplementary Figures

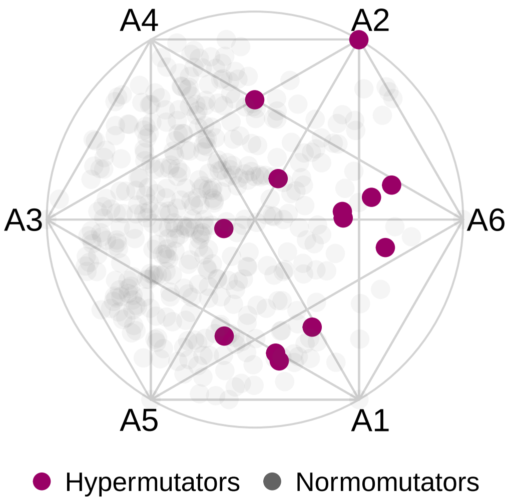

Supplementary Figure 1. Hypermutators versus normomutators

Supplementary figure 1, related to Figure 4, shows that while hypermutators do associate with 'adapted' archetypes, many other isolates are also present in the same simplex region despite not being hypermutators. Thus, hypermutators do not alone drive the extreme phenotypes defining the simplex boundaries, and normomutators are capable of producing similar phenotypes.

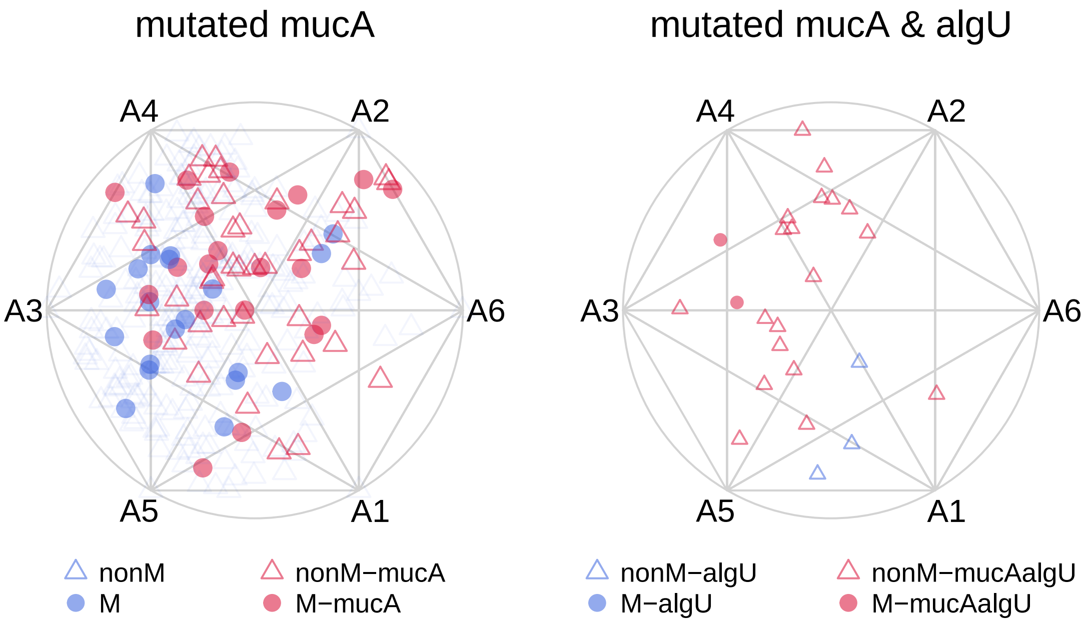

Supplementary Figure 2, related to Figure 5. mucA and algU mutants
We identify isolates by mutation (color) and mucoidity (shape), dimming all non-mucoid mucA WT isolates for improved clarity of the other groups. While most algU mutants are nonmucoid as expected, the relationship between mucA mutants and mucoidity is far less clear. Furthermore, there is no obvious archetypal association with any combination of mutations in this regulatory system, indicating no pattern or convergence among the phenotypes we tested.

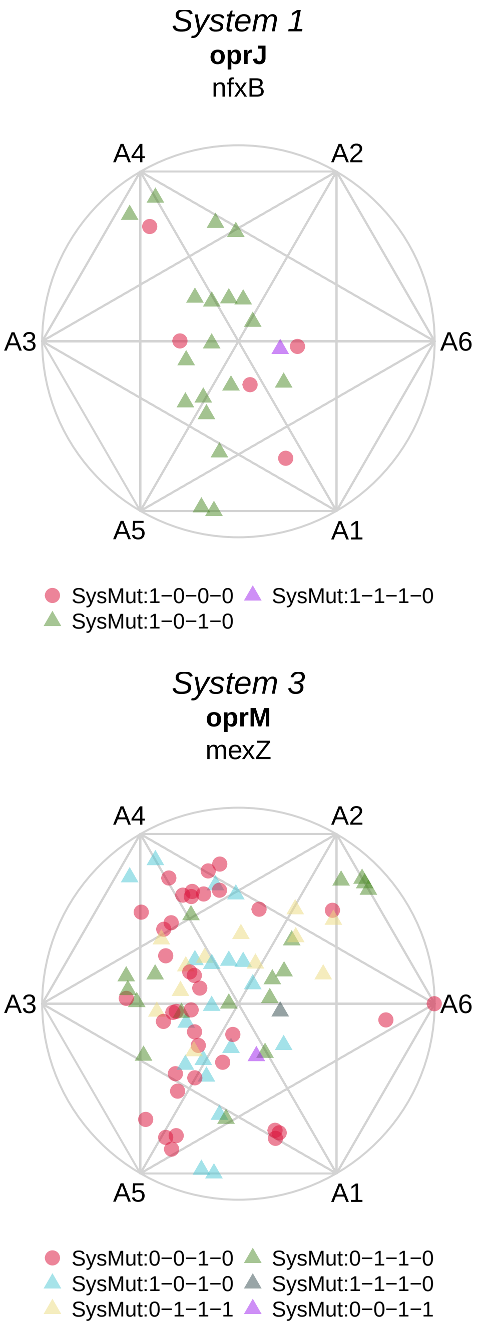

Supplementary Figure 3, related to Figure 5. mexZ mutants and drug efflux pumps

Because so many isolates had a mexZ mutation, we evaluated mexZ mutations in isolation and in combination with other regulators of drug efflux pump systems anchored by oprJ, oprM, and oprN. We separate nalC, nalD, and mexR from mexZ based on their regulation of mexAB versus mexXY in conjunction with oprM pump function. Figure S3 shows isolates with mutations affecting a particular efflux system's regulators (in red) as well as double and triple system hits that also impact that system indicated by the vectors in the legend. For example, the green triangles of the System 1 simplex plot represent isolates with mutations in system 1 regulator nfxB as well as a mutation in system 3 regulator mexZ. The single purple triangle indicates an isolate with mutations in the regulators of efflux systems 1, 2, and 3. What this analysis shows is that there is strong selective pressure on these pump systems, to such a degree that there are many isolates with 3 systems hit, though of course isolates with single and double systems hit are more prevalent. This makes it difficult to discern specific archetypes associated with a given mutated efflux system. Isolates with a mexZ mutant in fact disperse quite broadly across the simplex visualization, with the only spatial delineation being an avoidance of archetype 3.

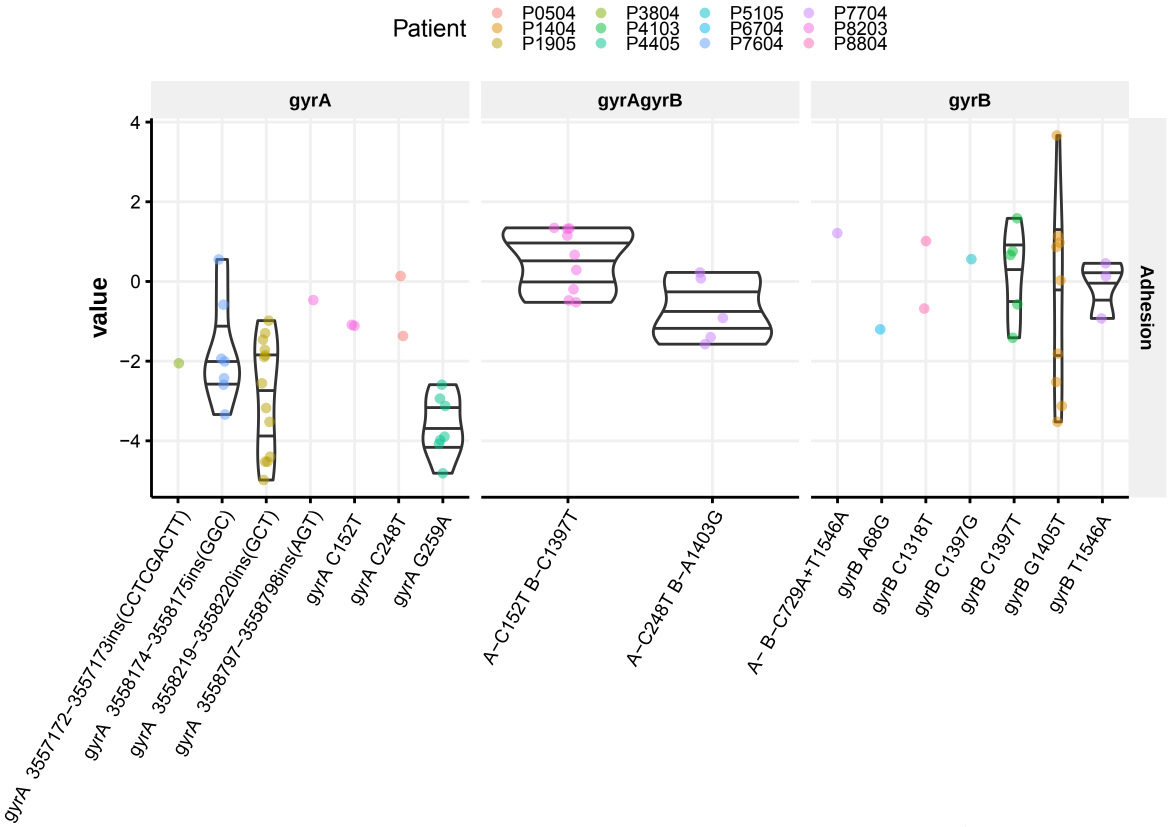

Supplementary Figure 4, related to Figure 5. Specific Mutations in gyrA/B by patient and adhesion After identifying a potential association between increased adhesion in isolates with gyrB mutations using both GAMMs and AA as illustrated in Figure 5, we decided to investigate whether gyrB mutations in isolation could increase adhesion in a WT background in comparison to gyrA mutation. Here, we compare measures of adhesion and patient origin for isolates with gyrA/B mutations to identify mutations associated with differential levels of adhesion. We selected gyrA C248T and G259A and gyrB C1397T and G1405T as appropriate representations of the range of adhesion measures linked to mutations in gyrA/B in our collection.

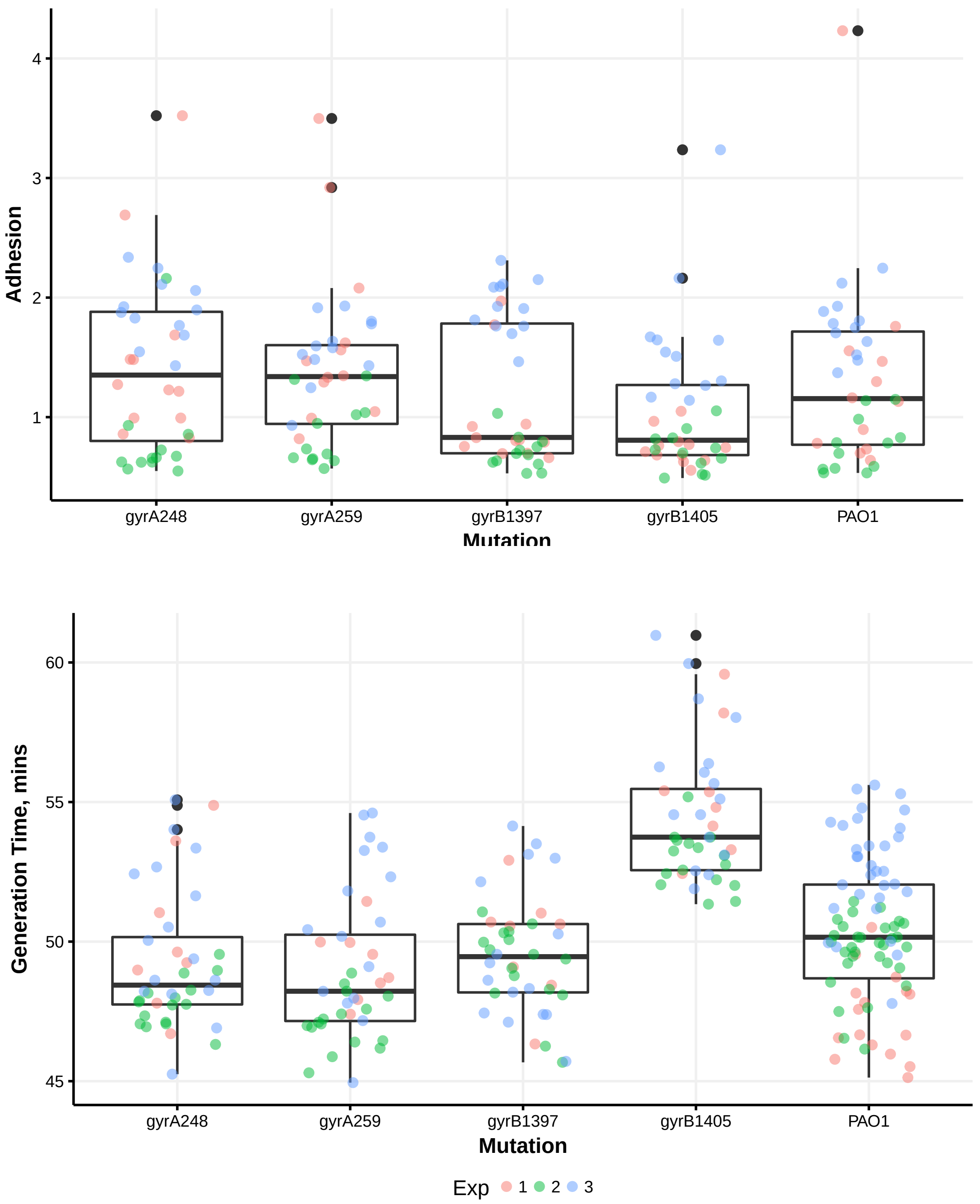

Supplementary Figure 5, related to Figure 5. Adhesion and generation time of gyrA/B mutants (PAO1) We moved our selected SNPs into a P. aeruginosa PAO1 background for screening of adhesion and growth rate in LB. After experiments to obtain 3 biological replicates with a minimum of 8 technical replicates each, we evaluated any phenotypic differences by analyzing the means of the technical replicates using one-way ANOVA with TukeyHSD. We saw a small but significant defect in growth rate due to the G1405T in comparison to the three other mutations and PAO1, but no difference in adhesion.

We include the summaries of our analysis of variance below using one-way ANOVA with Tukey's 'Honest Significant Difference' Method.

```
##             Df Sum Sq Mean Sq F value Pr(>F)
## Mutation     4 0.2684 0.06709   0.233  0.913
## Residuals   10 2.8786 0.28786

##   Tukey multiple comparisons of means
##     95% family-wise confidence level
##
## Fit: aov(formula = Adhesion ~ Mutation, data = adhDR)
##
## $Mutation
##                         diff       lwr      upr     p adj
## gyrA259-gyrA248   -0.04228223 -1.484010 1.399446 0.9999765
## gyrB1397-gyrA248  -0.20647903 -1.648207 1.235249 0.9883563
## gyrB1405-gyrA248  -0.37460897 -1.816337 1.067119 0.9067548
## PAO1-gyrA248      -0.10775541 -1.549484 1.333973 0.9990431
## gyrB1397-gyrA259  -0.16419680 -1.605925 1.277531 0.9950981
## gyrB1405-gyrA259  -0.33232674 -1.774055 1.109402 0.9368499
## PAO1-gyrA259      -0.06547318 -1.507201 1.376255 0.9998663
## gyrB1405-gyrB1397 -0.16812994 -1.609858 1.273598 0.9946345
## PAO1-gyrB1397      0.09872362 -1.343005 1.540452 0.9993215
## PAO1-gyrB1405      0.26685356 -1.174875 1.708582 0.9703572

##             Df Sum Sq Mean Sq F value Pr(>F)
## Mutation     4  65.61   16.40   5.636 0.0122 *
## Residuals   10  29.10    2.91
## ---
## Signif. codes:  0 '***' 0.001 '**' 0.01 '*' 0.05 '.' 0.1 ' ' 1

##   Tukey multiple comparisons of means
##     95% family-wise confidence level
##
## Fit: aov(formula = GenTime ~ Mutation, data = gyrDR)
##
## $Mutation
##                         diff        lwr        upr     p adj
## gyrA259-gyrA248   -0.4246656 -5.0088939  4.1595626 0.9977883
## gyrB1397-gyrA248   0.1698721 -4.4143561  4.7541003 0.9999404
## gyrB1405-gyrA248   5.2286783  0.6444501  9.8129065 0.0243988
## PAO1-gyrA248       0.4466276 -4.1376006  5.0308559 0.9973111
## gyrB1397-gyrA259   0.5945378 -3.9896904  5.1787660 0.9919712
## gyrB1405-gyrA259   5.6533440  1.0691158 10.2375722 0.0152799
## PAO1-gyrA259       0.8712933 -3.7129349  5.4555215 0.9674425
## gyrB1405-gyrB1397  5.0588062  0.4745780  9.6430344 0.0294641
## PAO1-gyrB1397      0.2767555 -4.3074727  4.8609837 0.9995871
## PAO1-gyrB1405     -4.7820507 -9.3662789 -0.1978225 0.0401037
```

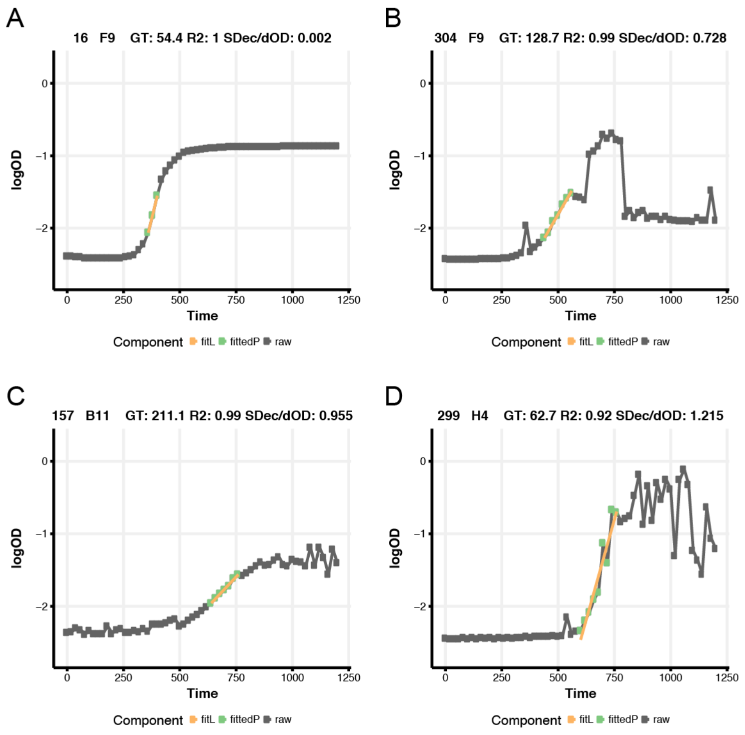

Supplementary Figure 6. Example growth curves

We developed our aggregation metric based on the idea that aggregates would disrupt the OD readings of growing cultures in an erratic way (via floating in and out of the measured well region). The binary method of estimating aggregation relied on the presence of both visible aggregates in cultures in microtiter wells and noise/fluctuations in the corresponding growth curve. The original binary metric also accounted for some degree of stochasticity - if 3 of 4 technical replicates showed aggregation, they were also marked as aggregating. This means that if fewer showed aggregation, they were not noted. To attempt to standardize this measurement via automation as well as obtain a continuous value for use in archetype analysis, we make the assumption that the noise in growth curves is reflective of aggregation alone, and that cases where noise is present without visual aggregation is simply reflective of less developed aggregates, which implies more of a development continuum. We show examples of the types of growth curve noise we see in the below figure. In these plots, we also show our method of growth curve fitting, as indicated by the green points and yellow fit line from which we derive growth rate and generation time (GT, in minutes) noted at the top of the graph. We include this information to show that we see aggregation at all speeds of growth (Curves B-D), and these curves are representative of many of the strains during growth in ASM. Furthermore, blanks of ASM (not shown) do not show noise in OD readings taken over the same time period as controls. Curve A shows a clean growth curve of one replicate of strain 16 (well F9) in Artificial Sputum Media (ASM). Regarding aggregation, we theorize that that we have cases of both large aggregates that move into the read region for a period and then disappear (Curve B and D) as well as smaller, more prolific aggregates that induce a lower level of continuous noise (Curve C). Our noise metric that we used to estimate these aggregation patterns is shown as SDec/dOD, i.e. sum of the decrease in OD over differential OD across the curve.

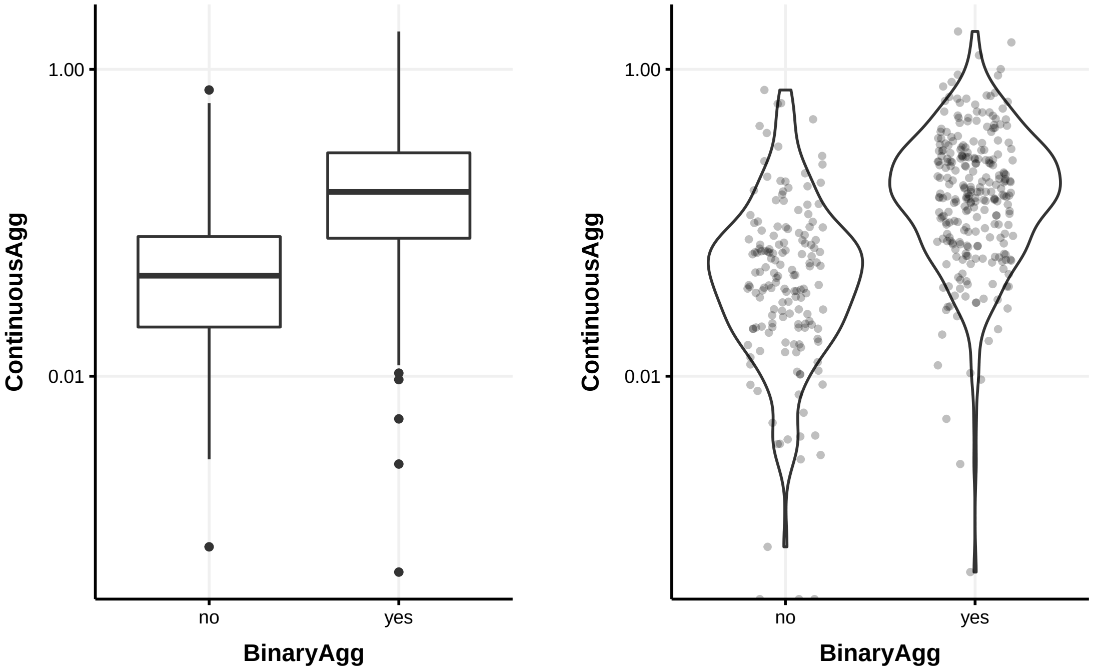

Supplementary Figure 7. Development of an aggregation metric
When we compare our aggregation metric with the binary aggregation measures assessed visually during growth curve experiments, we see that a higher SDec/dOD corresponds to visible aggregation. However, there is overlap between the categories, particularly at average continuous values of SDec/dOD. We are not sure whether this represents a higher degree of accuracy in our quantitative, automated metric, which may capture levels of aggregation that are difficult to see visually in the microtiter wells, whether it is a factor of averaging SDec/dOD over strain replicates that may not all be aggregating (or aggregating to a similar degree), or the metric needs to be improved. However, the metric appears more effective at its extreme values, and these values are what really contribute to meaningful separation in archetype analysis. We plan to continue improving this metric in the future, but for now it is our best available estimate of aggregation using continuous values. Furthermore, GAMMs analysis shows that changes in our continuous aggregation metric SDec/dOD have a significant positive correlation with adhesion over time, further supporting a relationship between this metric and an adherence phenotype.

Supplementary Note 1 - Archetypal Analysis

Jennifer Bartell

Archetype analysis

Archetype analysis is a data decomposition technique that maps samples according to their relationship to extreme points in the dataset trait landscape. We have used the 'archetypes' R package by Eugster, Leisch, and Seth (updated in 2014) to perform this analysis. (https://cran.r-project.org/web/packages/archetypes/archetypes.pdf (https://cran.r-project.org/web/packages/archetypes/archetypes.pdf)).

Archetype analysis involves representing observations (here, clinical isolates) described by multiple measures (here, phenotypic traits), as convex combinations of 'archetypes' representing the extremes of the multi-trait observation space (i.e. the corners of the minimal polyhedron that encapsulates this space). To do so, one solves a nonlinear least squares problem using an alternating minimizing algorithm.

Mathematically, a dataset *X* is represented as an *m* x *n* matrix of *m* observations described by *n* traits. Via archetype analysis, we find solution matrix *Z* containing the characteristic trait values for *k* archetypes with trait values for *n* traits. *k* must be specified a priori and selection should be optimized according to RSS of fit at varying *k* numbers.

To obtain *Z* and α, an *n* by *k* matrix containing the archetype coefficients for each observation which are 0 and sum to 1, the dataset is fit using alternating minimizations of the following equations:

$$RSS = ||X - \alpha Z^{\intercal}||_2 \tag{1}$$

$$Z = X^{\intercal}\beta \tag{2}$$

β represents the *n* by *k* coefficients of the dataset, which also are 0 and sum to 1, enforcing that the archetypes are also convex combinations of the dataset.

In summary, the algorithm calculates an initial set of archetypes and then iteratively alternates between finding the archetypes that best fit the data, and the data coefficients that best fit the archetypes, ultimately converging on an optimized representation of both by solving a series of linear equations and convex least squares problems. Multiple solutions are possible as convergence is dependent on the quality of archetypes used to initiate the solve and results must also be assessed with respect to avoiding local minima, so repeated fits using different initializations must be compared. This method is also reliant on the extreme measures in the dataset, so an assessment of outliers and their impact is necessary.

Overview

This document includes code necessary to repeat our archetype analysis, model evaluation and fit assessments, and an explanation of our archetype ordering method. We referred to Thøgersen et al. and Fernandez et al. in the design of an appropriate assessment method for the final model, relying on Explained Sample Variance (ESV) as an additional 'best fit' metric for assessing isolate-specific fit accuracy and uniformity as well as different fit visualizations as shown in this document. We added a model optimization approach to ensure repeatable model convergence and avoid local minima by aligned comparison of the top 10 'best fit' models of 500 independent solves.

Primary References

Eugster, M. J. A. & Leisch, F. (2009). From Spider-Man to hero: Archetypal analysis in R. J. Stat. Softw. 30, 1–23. https://doi.org/10.18637/jss.v030.i08 (https://doi.org/10.18637/jss.v030.i08)

Eugster, M. J. A., & Leisch, F. (2011). Weighted and robust archetypal analysis. Computational Statistics and Data Analysis, 55(3), 1215–1225. https://doi.org/10.1016/j.csda.2010.10.017 (https://doi.org/10.1016/j.csda.2010.10.017)

Thøgersen, J. C., Mørup, M., Damkiær, S., Molin, S., & Jelsbak, L. (2013). Archetypal analysis of diverse Pseudomonas aeruginosa transcriptomes reveals adaptation in cystic fibrosis airways. BMC Bioinformatics, 14, 279. https://doi.org/10.1186/1471-2105-14-279 (https://doi.org/10.1186/1471-2105-14-279)

Fernandez, M., Wilson, H., & Barnard, A. S. (2017). Impact of Distributions on the Archetypes and Prototypes in Heterogenous Nanoparticle Ensembles. Nanoscale, 9, 832–843. https://doi.org/10.1039/C6NR07102C (https://doi.org/10.1039/C6NR07102C)

Initialization

```
### save directory
sdir="~/Archetypes/fitchecks/"
dir.create(sdir, showWarnings = FALSE)

### loading libraries

library(gdata)
library(stringr)
library(tidyverse)
library(tibble)
library(readxl)
library(plyr)
library(ggthemes)
library(reshape2)
library(archetypes)
library(RColorBrewer)
library(GGally)
library(outliers)
library(ggpubr)
library(grid)
library(gridBase)
library(gridExtra)
library(factoextra)
library(gplots)
library(knitr)
library(cluster)
library(lubridate)

### custom functions used

#code from tutorial_archetypes_prototypes_SiQD_ensembles.r, Nanoscale. Royal Society of Chemistry 2016.
#Function to find the closest structures to the cluster centroids #or archetypes parameters
getSimilarToVector <- function(archetype,examples_coord,number){
  diff <- matrix(nrow=nrow(archetype),ncol=nrow(examples_coord))
  arch_close <- matrix(nrow=nrow(archetype),ncol=number)
  for (i in 1:nrow(archetype)){
    for (j in 1:nrow(examples_coord)){
      diff[i,j] <- sqrt(sum((examples_coord[j,]- archetype[i,])^2))
    }
  arch_close[i,] <- order(diff[i,])[1:number] }
return(arch_close) }

lowerFn <- function(data, mapping, method = "lm", ...) {
  p <- ggplot(data = data, mapping = mapping) +
    geom_point(alpha=.3,size=.1) +
    geom_smooth(method = method, color = "red", size=.3)
  p
}

set.seed(1234)

#loading pheno DB
Pheno_orig<-read_csv("~/Dropbox/DTU_CfB/Archetypes/PhenoPaper/Data/DBforMods_current.csv")
nrowct<-nrow(Pheno_orig)
Pheno<-Pheno_orig
```

Choosing/Processing Variables for Archetype Analysis

We are starting our analysis with 443 isolates to evaluate, and 8 phenotypic variables that can be used to describe these isolates. Two of these measures are MICs for different antibiotics, while the other six are physiologic phenotypes such as growth, adhesion ability, and protease production.

```
Pheno_unsc<-subset(Pheno,select=c(
                          GR_LB,GR_ASM,aggASMavg,AdhesionN,#continuous
                            Protease,Mucoid, #discrete
                            azt,cip #abx
                            ))

colnames(Pheno_unsc)<-c('GRLB','GRASM','ASMagg','Adhesion','Protease','Mucoid','azt','cip')

Pheno_unsc$Protease<-as.factor(Pheno_unsc$Protease)
Pheno_unsc$Mucoid<-as.factor(Pheno_unsc$Mucoid)

pairs1<-ggpairs(Pheno_unsc,lower = list(continuous = wrap(lowerFn, method = "lm")),
                 upper = list(continuous = wrap("cor", size=3)),diag=list(continuous=wrap("barDiag"))) +
    theme_Publication()+theme(text=element_text(size=8))+labs(title='Raw Phenotypes')
print(pairs1)
```

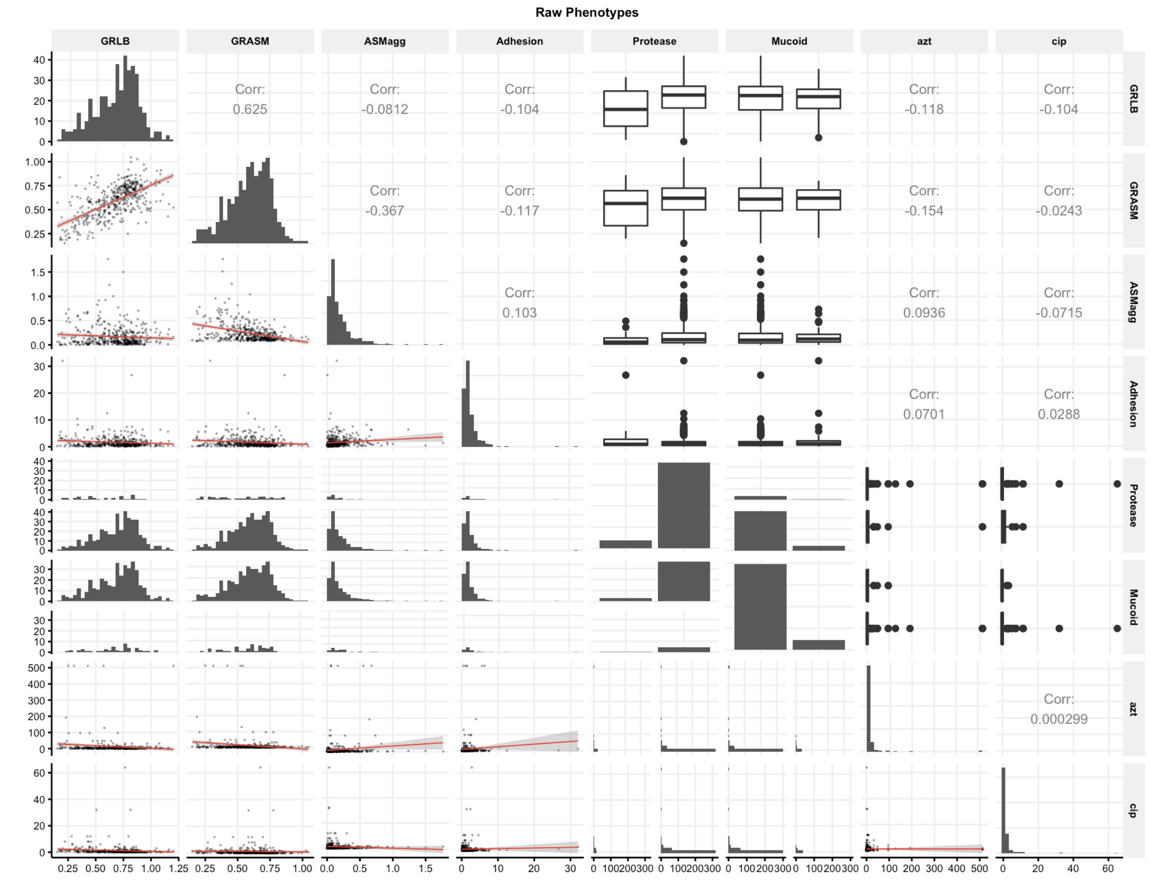

Fitting different classes of data (fx. continuous versus discrete versus binomial) using archetype analysis is not recommended. Therefore, for one variable, ASM aggregation, we use representative continuous variables derived from the noise in our growth curves as detailed in methods so that they are accessible for our archetype analysis approach. We remove Protease and Mucoid phenotype descripters as they are binary / discrete, and we do not have appropriate data for any conversion to a continuous measure at the moment. Therefore, we start our modeling with the six phenotypes that can be represented with continuous values or have at minimum a large continuum of discrete values (antibiotic resistance). Descriptive classifiers like singleton and evolved are also retained as they will be used as overlays on final archetype fits rather than informing the archetypes. Similarly, we can overlay Genotype, Patient, Isolate Age, and other classifiers.

Data scaling

One can use both scaled and unscaled data in archetype analysis, as one of its priorities is to preserve easily interpretable descriptions of the isolates. Therefore, we standardize the data by normalizing by PAO1 value (for all but antibiotic MICs) and then performing log2 transformation. For antibiotic resistance measures, we normalize by MIC breakpoint to enable cross-abx comparisons and then perform log2 transformation. The below figure shows the effect of our scaling steps on all variables, particularly visible in the histograms on the diagonal.

```
#log transforming resistance data (already normalized by EUCAST breakpoint MICs)
Pheno$aztNS<-log2(Pheno$aztN)
Pheno$cipNS<-log2(Pheno$cipN)

#Adhesion normalized it by PAO1 adhesion (2.53) and then log2 transformed
Pheno$AdhesionNS<-log2(Pheno$AdhesionN/2.53)

#PAO1 and PA14 do not aggregate and have very low but non-zero aggregation values, but other isolates have a valu
e of zero, so we add a correction factor of 0.01 to enable log2 transformation
Pheno$ASMaggNS<-Pheno$aggASMavg
PAO1aggm<-0.069776085
Pheno$ASMaggNS<-log2((Pheno$aggASMavg+.01)/(PAO1aggm+.01))
#hist(Pheno$ASMaggNS,breaks=30)

#normalize by WT growth rate
Pheno$GR_LBNS<-log2(Pheno$GR_LB/(log(2)/44.5728468*60))
Pheno$GR_ASMNS<-log2(Pheno$GR_ASM/(log(2)/48.81447217*60))

PhenSub_base<-subset(Pheno,select=c(
                          GR_LBNS,GR_ASMNS,ASMaggNS,AdhesionNS,#continuous
                            #Protease,Mucoid, #discrete
                            aztNS,cipNS #abx
                            ))

colnames(PhenSub_base)<-c('GRLB','GRASM','ASMagg','Adhesion','azt','cip')

#retain descriptors/classifiers in second dataframe
eval_data_base<-PhenSub_base
eval_data_base$ID<-Pheno$ID
eval_data_base$IageCT<-Pheno$IageCT
eval_data_base$DSJ<-Pheno$DSJ
eval_data_base$Genotype<-Pheno$Genotype
eval_data_base$Patient<-Pheno$Patient
eval_data_base$singleton<-Pheno$singleton
eval_data_base$evolved<-Pheno$evolved
eval_data_base$ULAW<-Pheno$ULAW
eval_data_base$hypermutator<-Pheno$hypermutator

arch_data_base<-PhenSub_base

pairs2<-ggpairs(arch_data_base,lower = list(continuous = wrap(lowerFn, method = "lm")),
                 upper = list(continuous = wrap("cor", size=3)),diag=list(continuous=wrap("barDiag"))) +
    theme_Publication()+theme(text=element_text(size=8))+labs(title='Continuous Scaled Phenotypes')
print(pairs2)
```

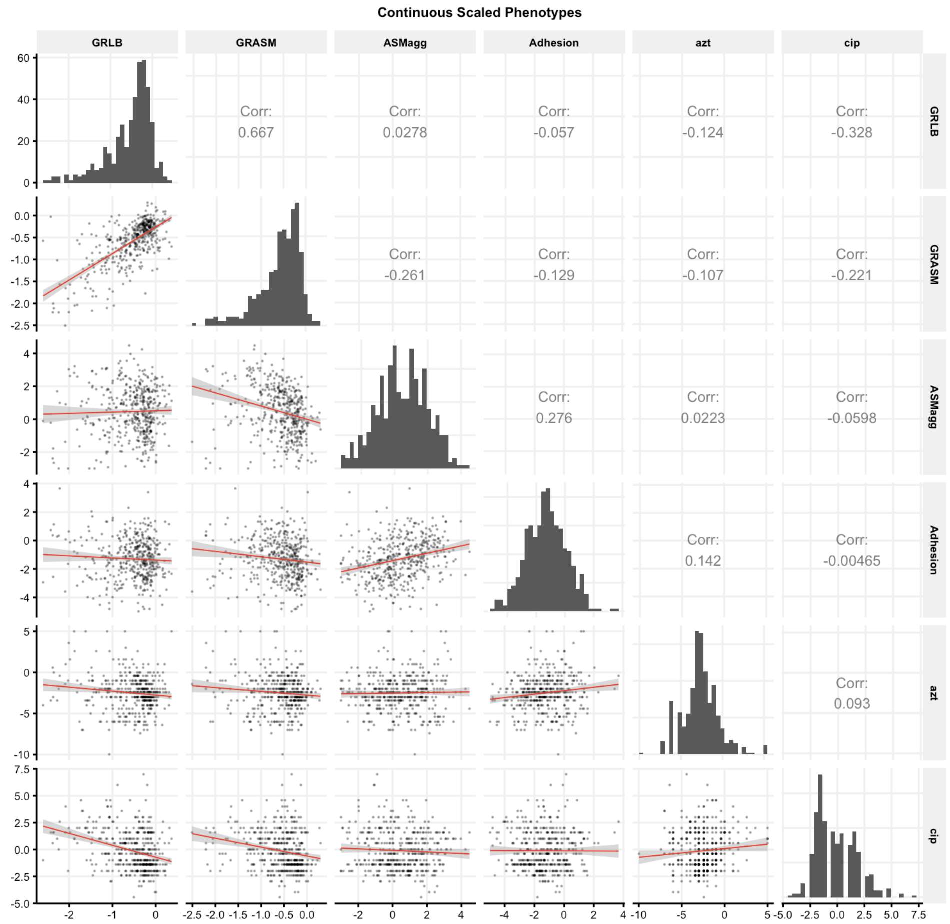

Archetype fitting - 5 phenotypes - robust fit

We can group our continuous phenotypes into growth, biofilm, and antibiotic resistance categories. We removed LB growth rate as LB and ASM growth rates are correlated substantially more than all other variable pairings. This resulted in the 5 reasonably independent phenotypes shown below:

growth-linked - growth rate in ASM (continuous)

biofilm-linked - adhesion (continous)

biofilm-linked - aggregation in ASM (continuous)

resistance-linked - Aztreonam susceptibility (semi-continous)

resistance-linked - Ciprofloxacin susceptibility (semi-continous)

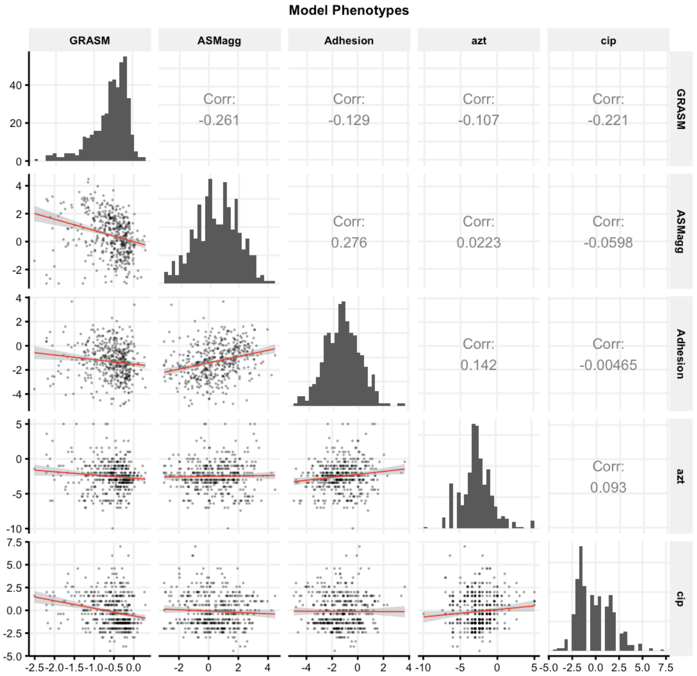

In fitting an archetype model to this data, we begin by stepping through fits of different archetype numbers to obtain a screeplot that allows us to choose the optimal number of archetypes to represent the data. However, despite setting a seed, we realized that the model fits rarely converged to an exactly repeatable set of archetype definitions using default solve parameters though all fit models suggested that 6 archetypes were an ideal representation of the data according to a screeplot check. To address this problem, we used the ‘robust’ archetype fitting technique which identifies and underweights outliers in the data, and ran 500 simulations of a 100 iteration fit of 6 archetypes to ensure repeatable convergence to the best fitting model based on evaluation of the archetype distributions and explained sample variance.

Below, we show a screeplot from 25 independent ‘robust’ archetype simulations using 50 iterations per step (with bars representing the standard deviation over the 25 simulations), which justifies that 6 archetypes are the ideal number to fit.

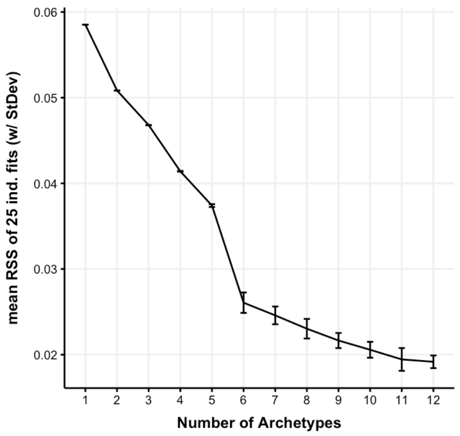

We ultimately run 500 simulations of a 100 step ‘robust’ fit to reproducibly obtain the best possible model based on the average of explained sample variance (ESV) calculated for each fitted isolate.

```
### commented to prevent accidental overwrite - code for running the 500 sims of 100 steps of the robust archetyp
e fit

# #
# ESVloop<-matrix(NA,ncol=2,nrow=500)
# for (ri in 1:500) {
#
#   rm(bfit,bfit_f,fitn)
#   set.seed(ri)
#   fitn<-stepArchetypes(data = arch_data_sc,k = 6, verbose = TRUE, nrep=100,method=robustArchetypes)
#   bfit_f<-bestModel(fitn)
#   #nmf<-paste('01robustloop5H_',as.character(ri),sep="")
#   nmf<-paste('robustloop100_',as.character(ri),sep="")
#   saveRDS(bfit_f, paste(sdir,"phenosubfitA6_Jan18_",nmf,".rds",sep=""))
#   #bfit_f<-readRDS(paste(sdir,"phenosubfitA6_Jan18_",nmf,".rds",sep=""))
#
#   rm(ESV,XCs,ESV_gri,ESV_agei)
#   XCs <- bfit_f$alphas %*% bfit_f$archetypes
#   ESV = (rowSums(arch_data_sc**2) - rowSums((arch_data_sc-XCs)**2))/rowSums(arch_data_sc**2)
#   ESV_gri<-order(eval_data$GRASM,decreasing = TRUE)
#   ESV_agei<-order(eval_data$IageCT,decreasing = FALSE)
#
#   bfitf_ESV80<-as.numeric(length(which(ESV>=.8)))/length(ESV)*100
#   bfitf_meanESV<-mean(ESV)
#   ESVloop[ri,1]<-bfitf_meanESV
#   ESVloop[ri,2]<-bfitf_ESV80
#
#  }
#
#    saveRDS(ESVloop,paste(sdir,"ESVloop_11Jan18_100steps_500solvesbestA6.rds",sep=""))
```

Explained sample variance

Here, we show the ESV results of our top model fits from the 500 independent solves of the 100 iteration robust fits using k=6 archetypes.

```
ESVloop<-readRDS(paste(sdir,"ESVloop_11Jan18_100steps_500solvesbestA6.rds",sep=""))

ESVl<-as.data.frame(ESVloop)
colnames(ESVl)<-c("bfitf_meanESV","bfitf_ESV80")
ESVl$bfitf_meanESV<-ESVl$bfitf_meanESV*100

ESVlm<-melt(ESVl)
esvp1<-ggplot(ESVlm) + geom_violin(aes(x=variable,y=value),trim=TRUE) + theme_Publication()
esvp1<-esvp1+xlab('')+ylab('Percentage')
print(esvp1)
```

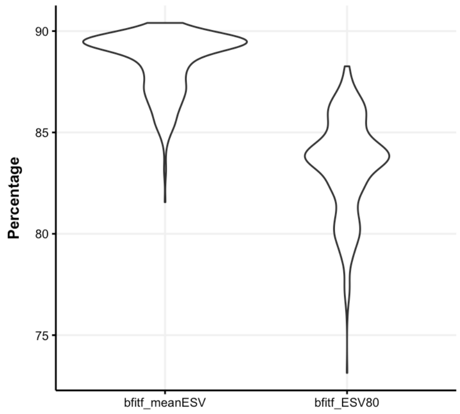


```
best10<-top_n(ESVl,10,bfitf_meanESV)
bestid<-which(ESVl$bfitf_meanESV %in% best10[,1])
ESVlp<-as.data.frame(ESVl[bestid,])
ESVlp$Model<-as.factor(bestid)
ESVlp<-subset(ESVlp,select=c('Model','bfitf_meanESV','bfitf_ESV80'))
kable(ESVlp,digits = 2,align="c",format="html",table.attr = "style='width:50%;'",caption = "Top 10 models by ESV"
,row.names=FALSE)
```

Top 10 models by ESV

Model	bfitf_meanESV	bfitf_ESV80
23	90.28	86.91
79	90.28	86.91
94	90.27	86.46
125	90.32	87.13
174	90.27	86.68

Model	bfitf_meanESV	bfitf_ESV80
177	90.39	87.13
219	90.28	86.68
228	90.40	86.91
313	90.31	87.13
396	90.32	87.13

The violin plot shows the distribution of the mean ESV of top models from each of the 500 simulations, showing that we cannot obtain a mean ESV higher than 90.4%. In investigating the characteristics of the top 10 models of the 500 saved models, which all have an ESV of at least 90%, we see that 9 of the 10 are essentially identical in their archetype (and isolate) components (see summary pdfs with archetype characteristics and representative simplex plots for reference - archetypes are ordered automatically by growth rate to enable comparison across simplex plots of each fit).

We chose the best model of the 9 highly similar fits based on both the highest mean ESV of the group and the percentage of isolates with an ESV greater than 80%. We show the explained sample variance data for each isolate in our optimal model (90.32 mean ESV), sorting the data by both isolate age and growth rate.

```
# the best fit achievable in 50k solves - very similar to other fits above 85% mean ESV
ri=bestid[4]
rm(bfit,bfit_f,fitn)
  set.seed(ri)
  #nmf<-paste('01robustloop5H_',as.character(ri),sep="")
  nmf<-paste('robustloop100_',as.character(ri),sep="")
  bfit_f<-readRDS(paste(sdir,"phenosubfitA6_Jan18_",nmf,".rds",sep=""))

rm(ESV,XCs,ESV_gri,ESV_agei)
  XCs <- bfit_f$alphas %*% bfit_f$archetypes
  ESV = (rowSums(arch_data_sc**2) - rowSums((arch_data_sc-XCs)**2))/rowSums(arch_data_sc**2)
  ESV_gri<-order(eval_data$GRASM,decreasing = TRUE)
  ESV_agei<-order(eval_data$IageCT,decreasing = FALSE)
  bfitf_ESV80<-round(as.numeric(length(which(ESV>=.8)))/length(ESV),digits=2)*100
  bfitf_meanESV<-mean(ESV)

   par(mfrow=c(3,1))
   par(mai=c(.6,.3,0,0))
    barplot(ESV,ylim=c(0,1.1),space=10,ylab = "Pheno Arch - Sample Explained Variance",xlab = "Isolates")
    par(mai=c(.6,.3,0,0))
    barplot(ESV[ESV_gri],space=10,ylim=c(0,1.1),ylab = "Pheno Arch - Sample Explained Variance",xlab = "Isolates
(fast to slow)")
    par(mai=c(.6,.3,0,0))
    barplot(ESV[ESV_agei],space=10,ylim=c(0,1.1),ylab = "Pheno Arch - Sample Explained Variance",xlab = "Isolates
(young to old)")
```

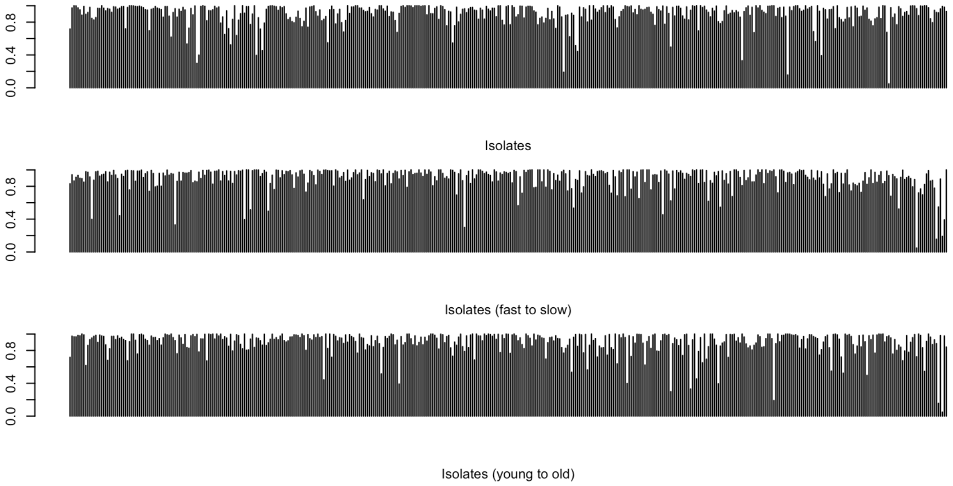

87% of the 443 isolates being fit have an explained variance of more than 80% for the 6 archetype model.

Parallel coordinate plot

We see that the 6 archetypes capture much of the data's extreme values using a parallel coordinates plot.

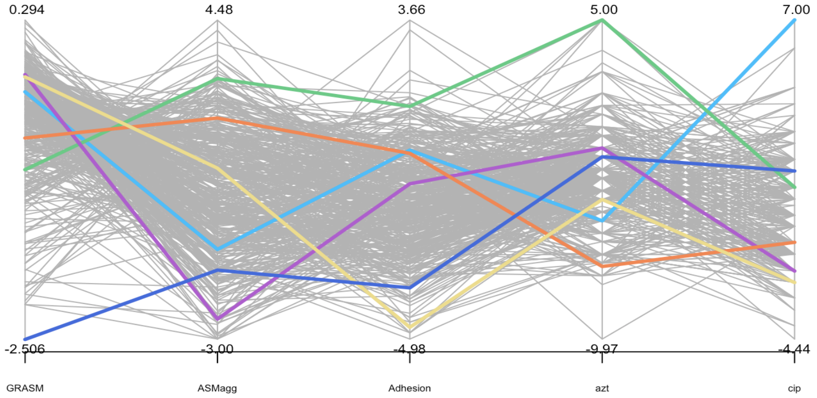

Convex hulls - 2D comparisons of archetype representation

We also use a grid of convex hull plots to evaluate the fits of the relationships between different phenotypes. The archetypes are the red points along the fit convex hull (red line) versus the original data (gray circles) and convex hull (transparent blue area). We want the archetype hull to capture as much of the original data as possible and for the archetypes to align with reasonable ‘extremes’ in the data. Archetype location is altered by isolate density as well as extreme values, though extreme artifacts are reduced when using the ‘robust’ archetype fit. Archetype locations that seem superfluous likely are driven by extremes in other dimensions of the fit (other variables). The below grids shows how the archetypes are distributed across all possible 2D combinations of the variables for the 6 archetype model. The ‘robust’ fit approach has reduced the power of outliers on archetype locations in comparison to normal archetype fits, while the main body of data density is captured within the convex hull.

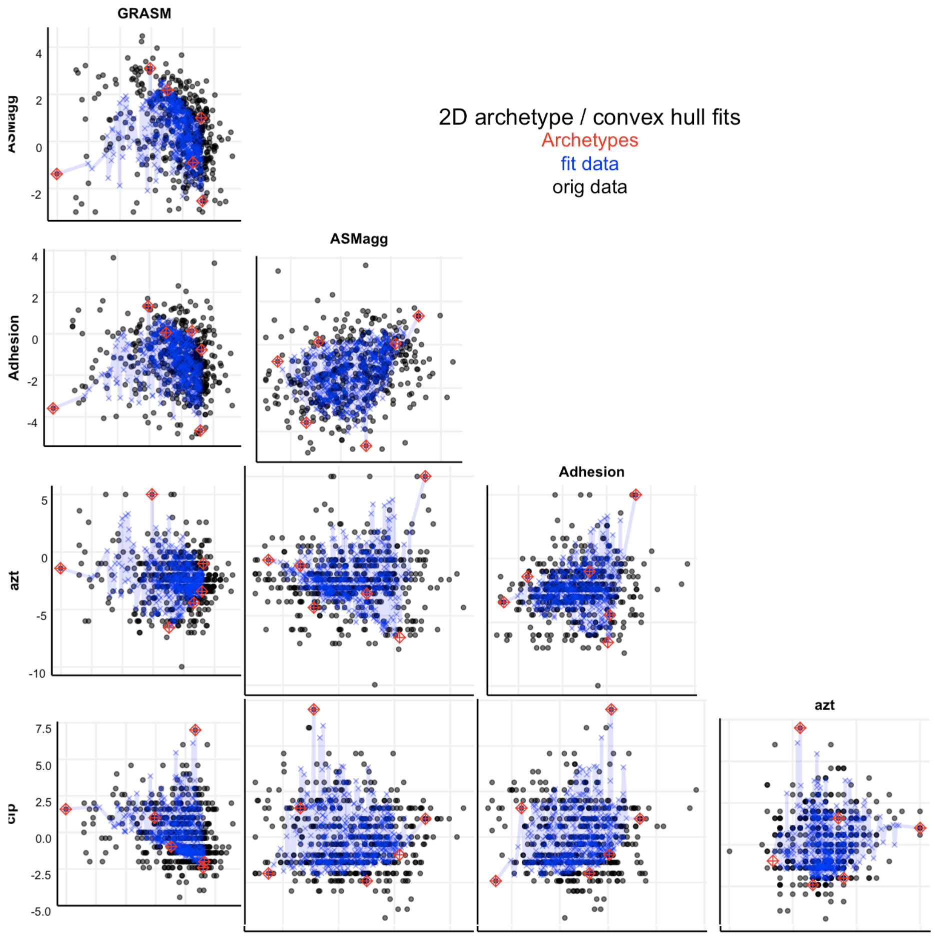

Archetype characteristics

Here, we evaluate the phenotypic characteristics of each archetype. We view this information by isolate grouping and phenotype grouping in the following two graphs. The first presentation allows us to more easily identify archetypes that we would expect to describe young versus old isolates, while the second allows us to quickly identify archetypes representing an extreme value in a particular phenotype and order archetypes by a particular phenotype.

In the first plot, we see that archetypes 3, 4, and 5 show WT levels of growth, while archetype 2 and particularly archetype 6 represent slow-growing isolates. Archetypes 3 and 5 most represent what we would hypothesize to be naive, unadapted isolates given their antibiotic susceptibility, reduced aggregation and reduced adhesion.

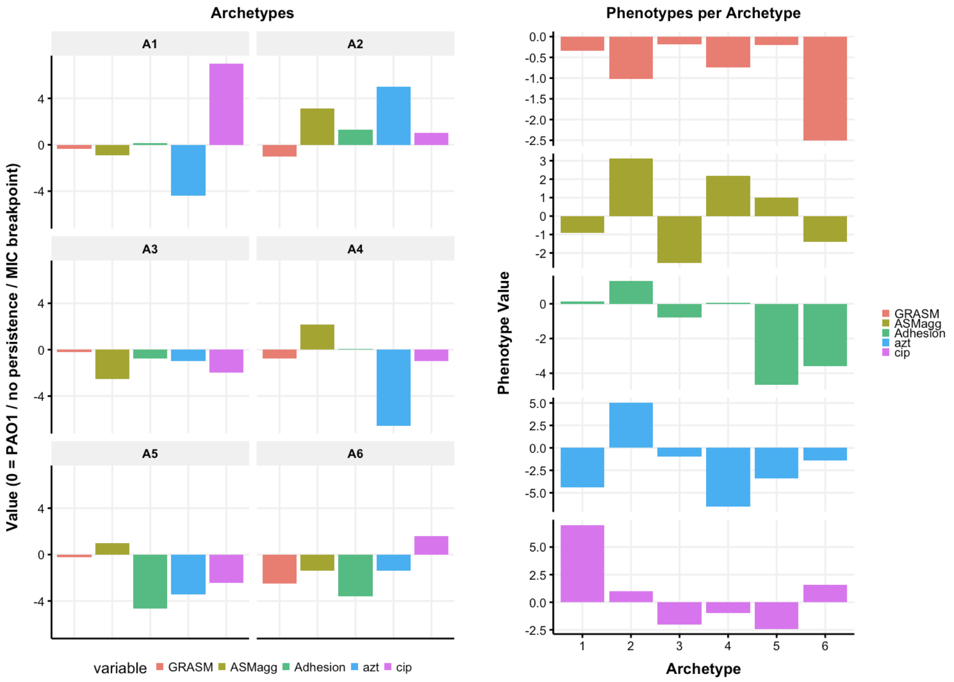

Using the second plot, we find the easiest way to evaluate the archetypes is to correlate the highest 2 and lowest two values of each phenotype to their respective archetypes. We also see that the 6 archetypes still allow for contrasts in the direction of relationships among the data. For example, 'slow growing' archetypes are paired with both high and low biofilm (both aggregation and adhesion) and resistance (cip vs azt) values. Ultimately the archetypes appear to enable many different phenotypic combinations, and the ultimate importance of each archetype can only be determined by evaluating the number of isolates it appears to strongly describe.

Isolate distribution among archetypes

The below heatmap shows both archetype linkage and how each isolate is described by the archetypes (ie what does each archetype contribute in the loadings of each isolate).

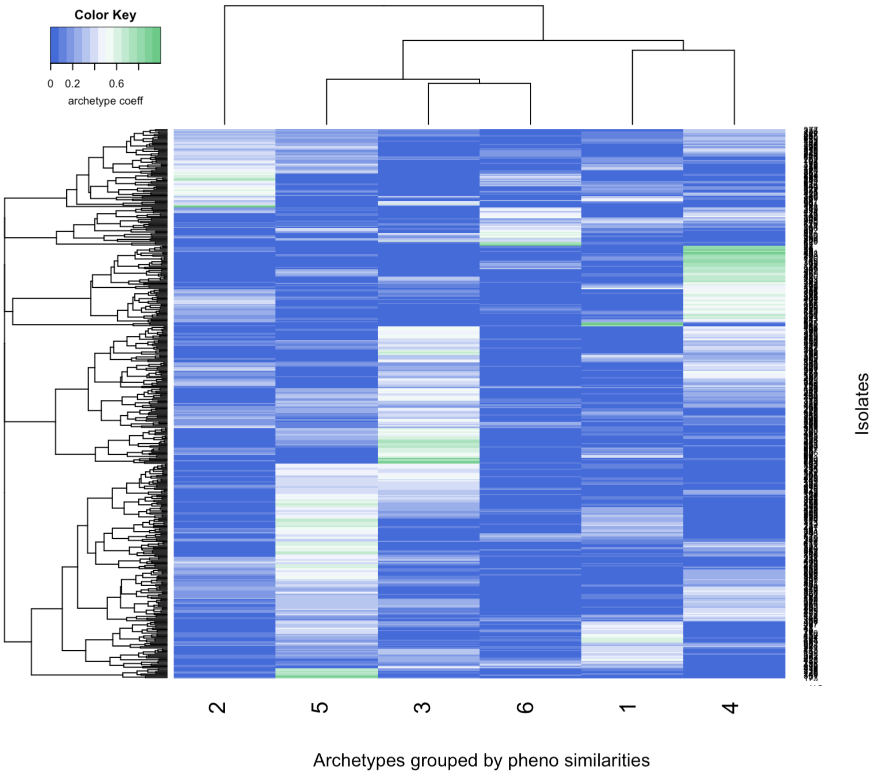

We can see that archetype 4 contributes a substantial loading (coefficient over 75%) to the most isolates by far, while archetype 5 contributes loading (coefficient over 25%) to the most isolates overall. Archetype 3 accounts for nearly as many isolates overall as archetypes 4 and 5. However, most isolates appear to be blends of at least a few different archetypes as indicated by the limited amount of green cells (equivalent to a coefficient of 1) in the heatmap. Archetype 6 contributes to the fewest isolates in total. The table below shows both the sum of contribution to all isolates as well as mean and standard deviation. This highlights the degree of separation between archetype roles and, for example, the significant role of Archetypes 4 and 5. The last three columns show the number of isolates that each archetype contributes more than 25%, 50%, and 75% in representation, showing that every archetype contributes substantially to at least one isolate's description. However, while 189 isolates are defined by at least 50% of one archetype, only 28 are defined by more than 75%. The following violin plot further indicates the distribution of archetype representation among isolates from a density standpoint.

Summary of Isolate Archetype Contributions

Std	Sum	Mean	Archetype	Over25pct	Over50pct	Over75pct
0.16	54.64	0.12	1	81	13	2
0.16	63.15	0.14	2	102	16	1
0.21	90.44	0.20	3	164	48	5
0.23	98.50	0.22	4	180	53	15
0.20	100.55	0.23	5	182	48	3
0.14	35.29	0.08	6	48	11	2

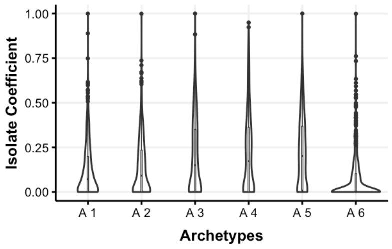

Simplex plots of isolate distribution

We would like to easily visualize relationships between isolates and the archetypes in a spatial way, and use simplex plots to do so. These plots project isolates according to their similarity to the prototypical archetypes defined by the above analysis. The first two plots show the general distribution of the isolates among the archetypes, as well as a projected directional connection of each isolate to archetypes contributing to its loading vector. Every archetype has at least one isolate representing its particular characteristics exactly. Contributions viewed by isolate direction appear large and unidirectional along the periphery of the data (ie near the archetypes, each isolate is mainly described by a single archetype) while multiple vectors grow in a more balanced way at the center of the plot. However, this plot is fairly difficult to figure out and we will focus on overlays of characteristic data as a better way to evaluate isolate relationships. Finally, we overlay ESV values for each isolate to see if there is a particular pattern to poorly fit isolates, and we particularly highlight isolates with an ESV < 50%. We can see that few isolates are fit this poorly, and they seem to be randomly distributed in the center of the simplex plot. The table below the plots shows the characteristics of these poorly fitting isolates, and no clear pattern emerges from the phenotype values. However, several of the isolates are from the same lineages.

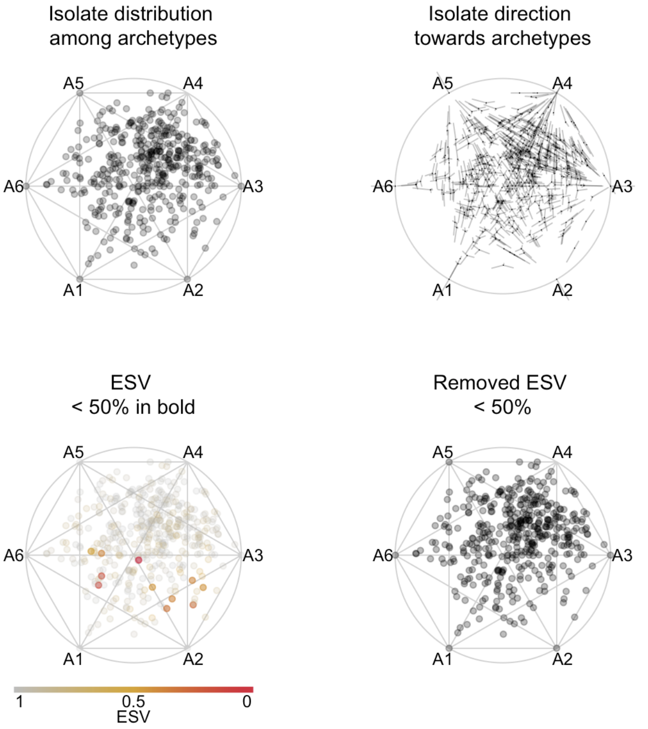

The simplex visualization is easy to read considering the dimensionality at play, but its simplicity does encourage biased interpretation by implying that each archetype is equidistant. Furthermore, archetypes are not necessarily ordered according to the similarity of their characteristics. For all following plots, we have reordered the archetypes according to growth rate such that maximum growth rate and minimum

growth rate are on opposing sides of the simplex plot. At minimum, the archetypes must be ordered in some fashion, as the plots are otherwise always structured from A1 to A6 regardless of the actual value of each archetype, and which makes comparison between model fits very difficult. After inspection of archetype values, we also implemented a position swap of Archetypes 1 and 4 in the simplex plot to improve alignment of cip phenotype without altering the growth alignment substantially. So, our ordering improves visual reproducibility between model fits via growth ordering and structures the archetypes in an interpretable way via the second cip-based ordering. We may be inducing some bias into our analysis in this manner, but we have shown via our GAM models that there is a clear change in growth rate over time across all of our data, and we suspect that many other phenotypes may be related to this adaptive trajectory. We have found that if we structure the archetypes in such a way, it is vastly easier to interpret meaningful changes in the other phenotypes in addition to growth.

To support that the archetypes are accurately representative of data extremes and evaluate how to order them in a meaningful way, we projected them on a principal component analysis of the phenotype data used in archetypal analysis.

PCA assessment of archetype order

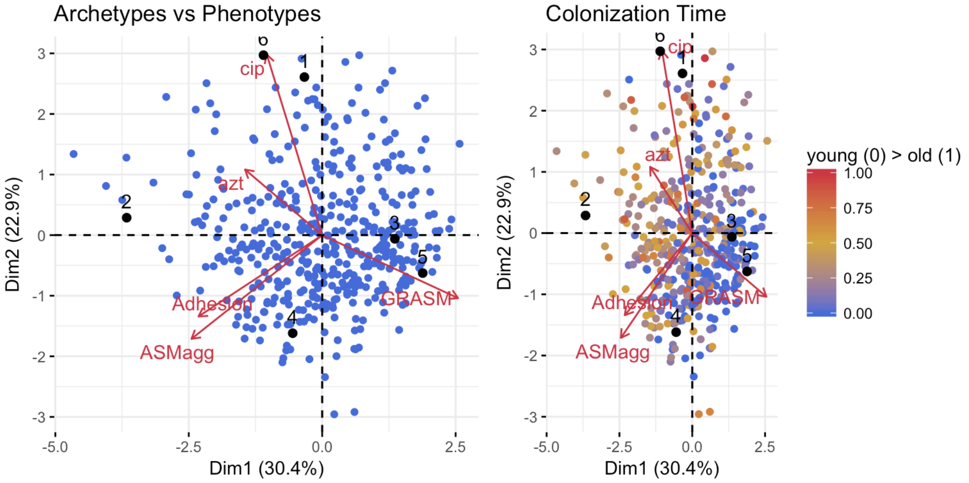

We see that the archetypes are positioned close to the boundaries of what appears to be continuous data (versus clusters of similar isolates). This, we believe, is a further argument for using archetype analysis as our ordination technique. Remarkably, the archetypes are also ordered almost identically to the way that we intuitively ordered them based on our theories about driving phenotype adaptation and GAMM models. We have switched the order of A3 and A5 as technically A3 has the fastest growth rate, which was our primary automated ordering phenotype, but these two archetypes are still placed quite close in the PCA plot.

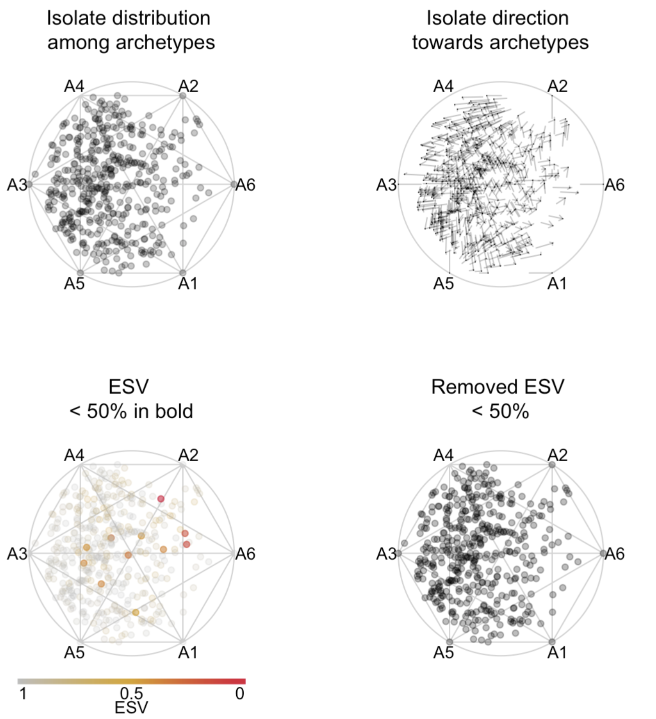

Evaluating isolate simplex distributions by phenotype

We overlay each phenotype involved in building the model to assess the degree of distribution / clustering versus the archetype definitions. This is a good way to confirm the model fit and get a feel for variation in the data. We can see an obvious continuum from low to high values for ASM growth rate and cip susceptibility. Order in the distributions of aztreonam susceptibility, ASM aggregation, and adhesion are less clearly demarcated. These results are similar to those from the 2D generalized additive models also presented in this work.

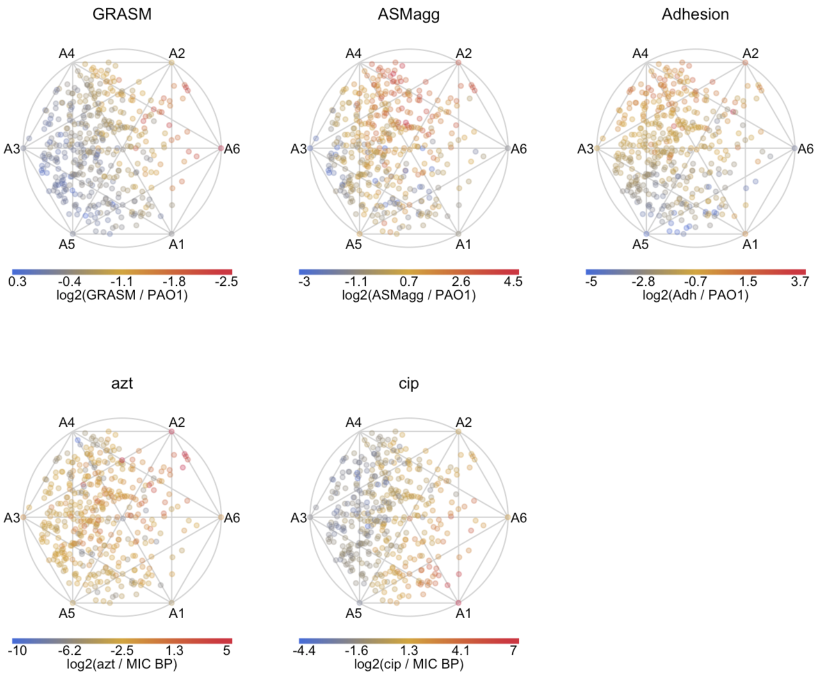

We next evaluate the isolate distributions using descriptive variables that were not included as a model factor. We start with isolate age by genotype (where time 0 is the first found isolate in a clonetype), the isolates we are positive are the first collected in a patient (and thus representing true adaptive starting points), and hypermutators. We see that young isolates broadly cluster near 'naive' high growth rates (A3 and 5), and older isolates are distributed near slow growing, resistant archetypes (A2, 6, and 1). Our true first isolates cluster near 'naive' archetypes, while the hypermutators cluster near the 'adapted' archetypes. The hypermutators also appear relatively early in infection while inducing an 'adapted' phenotype, which explains some of the noise in the age by genotype simplex.

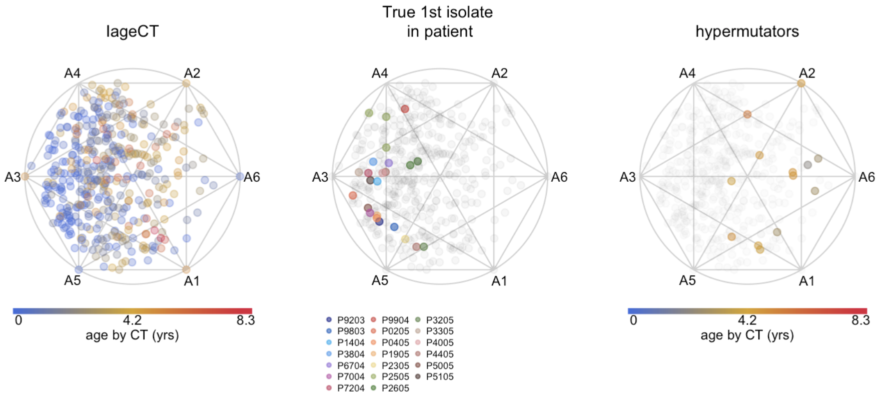

In the below panel of simplex plots, we compare isolate age (by genotype), growth data, and ciprofloxacin susceptibility measurements side by side. The color scheme is oriented such that red is indicative of the expected 'evolved' state while blue is indicative of an expected 'naive' state in all graphs, and yellow indicates an intermediate value. Thus you can see that young isolates are generally located around archetypes 4, 3, and 5, and these same archetypes best describe fast growing isolates in the next graph. The localization shifts slightly away from archetype 5 and towards archetypes 3 and 4 while indicating isolates susceptible to ciprofloxacin, but the same naive side of the plot is still shaded blue. Interestingly, the first plot does show older isolates mixed in with the majority young isolates on the upper left of the plot, but there is much less mixing of slower growing isolates with the isolates growing near WT levels or between ciprofloxacin susceptible and resistant isolates. Given that the second two plots are being overlaid with data used to develop the archetypes, it is not suspicious that we see the most irregular distribution by age. Instead, the division we see in the first plot is a good confirmation that the approach is working to appropriately separate isolates in a useful and unbiased way by phenotype.

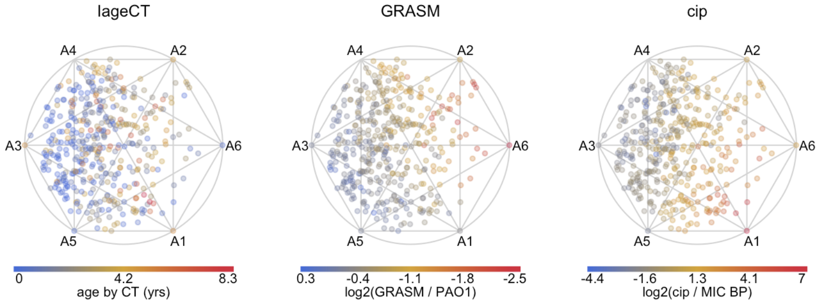

Supplementary Note 2 - Generalised additive mixed models

Lea Sommer

General introduction to this document and Generalized Additive Models (GAMs)

This document includes information on how the analysis with Generalized Additive Mixed Models (GAMMs) has been carried out.

To ease into what a GAMM is we start with Generalised Linear Models (GLMs). They are an extension of 'regular' linear regression. That is, the 'Generalised' part allows for the incorporation of different kinds of data other than continuous (factorial, binary, etc.) as the response variable and are thus more flexible, -this also goes for the 'Generalised' part of the GAMs.

The 'additive' part of GAMs as compared to the 'linear' part of GLMs has to do with the actual setup of the model. GAMs are semi-parametric extensions of GLMs in the way that a GAM can end up approximating a GLM. The special part of the GAM is that it does not assume a linear relationship between the variables, the only assumption is that the variables are additive and that their functions are smooth, i.e. the interaction will end up being a smooth line.

When introducing the 'mixed' part and making the GAM a GAMM is when we introduce the addition of random factors (this can also be done for GLMs as they then become GLMMs).

For further reading about GLM, GAM, GAMM, and GLMM we refer to the book "Generalized additive models, 2nd edition" by Simon N. Wood, who is also the author of the R package *mgcv* which we are using for the GAMM models used here.

Data/variable explanation

A short introduction to the variable names can be found in the table legend of Supplementary Table S1.

Summary of results

This section will show a summary of what the analyses found further down in this document show. We are aware of the risk of overfitting our data when employing GAMMs, and sought to minimize this risk via restricted maximum likelihood (REML) estimation. We alse use a feature reduction approach to avoid assuming any 'cause and effect' relationships between our variables

In brief, the analyses have been carried out by analysing the phenotypes one-to-one to find significant interactions determined by a p-value below 0.05, based on Wald-type tests as described in the papers:

Wood, S. N. Generalized additive models: an introduction with R.

Wood, S. N., Pya, N. & Säfken, B. Smoothing Parameter and Model Selection for General Smooth Models. J. Am. Stat. Assoc. 111, 1548–1563 (2016).

Wood, S. N. Fast stable restricted maximum likelihood and marginal likelihood estimation of semiparametric generalized linear models. J. R. Stat. Soc. Ser. B (Statistical Methodol. 73, 3–36 (2011).

Wood, S. N. On p-values for smooth components of an extended generalized additive model. Biometrika 100, 221–228 (2013).

In the one-to-one GAMMs IageCT (time since initial colonisation by the clone type) is always included to take the longitudinal sampling of the isolates into account. Time has also been taken into account in the random factor of patient, where we have used a random smooth function. Because the one-to-one models have the same layout independently of the predictor variable, the specific code for the models are only shown for the one-to-one models with growth rate in artificial sputum medium (ASM) ("GR_ASM").

After having done the one-to-one analyses we continued with more complex models where all explanatory variables with a significant impact on the predictor (significance cut-off: 0.05) were included in the GAMM. In these models IageCT was only included if it had shown a significant impact on the predictor. The p-values of these analyses can be found in the second table below.

Diagnostic plots using qq-plots, histograms of residuals, and residual plots have been used to see if the model assumptions are upheld for all models. These are not shown for the one-to-one models but have been visually inspected and found adequate for all models included in this study and all models shown in this supplementary material. We show an example of the diagnostic plots for the first one-to-one model (see below), but we otherwise only show these plots for the extended models with multiple explanatory variables.

An example of a summary of a model is shown below for the first one-to-one model, but for the rest of the one-to-one models this is not shown, as these were only used for identifying which explanatory variables that should go into the extended GAMMs.

p-values of the one-to-one analyses

It should be noted that p-values of 2.0e-16 are as low as the program can get and thus could in theory be lower than this.

	**Continuous explanatory variables**							**Binary explanatory variables**		
**Predictor**	**IageCT**	**GR_ASM**	**GR_LB**	**aztN**	**cipN**	**AdhesionN**	**ASMaggN**	**Protease**	**Mucoid**	**hypermutator**
GR_ASM	**5.8e-07**	NA	**2e-16**	**0.2**	**0.4**	**0.03**	**8.7e-13**	**0.4**	**0.3**	**1**
GR_LB	**5.08e-07**	**2e-16**	NA	**0.04**	**0.3**	**0.3**	**0.1**	**0.08**	**0.9**	**0.5**
aztN	**0.2**	**0.9**	**0.2**	NA	**0.8**	**0.01**	**0.9**	**0.9**	**0.3**	**6e-04**
cipN	**4.9e-09**	**0.9**	**0.6**	**0.06**	NA	**0.7**	**0.1**	**0.2**	**0.1**	**0.02**
AdhesionN	**0.6**	**0.04**	**0.02**	**0.008**	**0.3**	NA	**3e-04**	**0.9**	**0.6**	**0.6**
ASMaggN	**0.4**	**1.2e-09**	**0.4**	**0.7**	**0.1**	**8.2e-05**	NA	**2e-05**	**1**	**0.1**

p-values of the extended models

NAs have been put in where variables have shown to be non-significant (p-values > 0.05) in the one-to-one analyses or if they have been removed from the bigger models because of model reduction steps, where they have been found to be non-significant as an explanatory variable when in combination with other explanatory variables.

	Continuous explanatory variables							Binary explanatory variables		
**Predictor**	**IageCT**	**GR_ASM**	**GR_LB**	**aztN**	**cipN**	**AdhesionN**	**ASMaggN**	**Protease**	**Mucoid**	**hypermutator**
GR_ASM	**0.007**	NA	**2e-16**	NA	NA	NA	**3.6e-15**	NA	NA	NA
GR_LB	**8e-04**	**2e-16**	NA	NA	NA	NA	NA	NA	NA	NA
atzN	NA	NA	NA	NA	NA	**0.01**	NA	NA	NA	**3e-04**
cipN	**1.1e-07**	NA	NA	NA	NA	NA	NA	NA	NA	**0.009**
AdhesionN	NA	NA	**0.01**	**0.01**	NA	NA	**2e-04**	NA	NA	NA
ASMaggN	NA	**1.3e-08**	NA	NA	NA	**0.004**	NA	**2.2e-05**	NA	NA

The specific deviance explained and the r^2 values for the final extended model can be found in the model summaries below.

NOTE: When an explanatory variable is not significant, the model have been rerun without this variable as a reduced model. The p-values shown are the values from the reduced models. The specific models can be identified below.

Packages used

tidyverse Hadley Wickham (2017). tidyverse: Easily Install and Load the 'Tidyverse'. R package version 1.2.1. https://CRAN.R-project.org/package=tidyverse (https://CRAN.R-project.org/package=tidyverse)

mgcv Wood, S.N. (2011) Fast stable restricted maximum likelihood and marginal likelihood estimation of semiparametric generalized linear models. Journal of the Royal Statistical Society (B) 73(1):3-36

itsadug van Rij J, Wieling M, Baayen R and van Rijn H (2017). "itsadug: Interpreting Time Series and Autocorrelated Data Using GAMMs." R package version 2.3.

ggthemes Jeffrey B. Arnold (2017). ggthemes: Extra Themes, Scales and Geoms for 'ggplot2'. R package version 3.4.0. https://CRAN.R-project.org/package=ggthemes (https://CRAN.R-project.org/package=ggthemes)

knitr Yihui Xie (2017). knitr: A General-Purpose Package for Dynamic Report Generation in R. R package version 1.17.

kableExtra Hao Zhu (2017). kableExtra: Construct Complex Table with 'kable' and Pipe Syntax. R package version 0.6.1. https://CRAN.R-project.org/package=kableExtra (https://CRAN.R-project.org/package=kableExtra)

Phenotypic analyses

For all phenotypes GAMMs were used to identify evolutional trends over time since infection. We correct for the patient origin and inconsistent sampling over time using a smooth random factor.

The data

```
## # A tibble: 4 x 7
##                  ID SeqID SRA_accNo Genotype Patient       Date IageCT
##               <chr> <chr>     <chr>   <fctr>  <fctr>     <date>  <dbl>
## 1 C03P6204I110909a     1 ERS402418     DK03   P6204 2009-11-09  0.000
## 2 C03P6204I062110a     2 ERS402543     DK03   P6204 2010-06-21  0.616
## 3 C03P6204I010511a     3 ERS402645     DK03   P6204 2011-01-05  1.156
## 4 C03P6204I111510a     4 ERS402755     DK03   P6204 2010-11-15  1.016
```

Singleton lineages (a total of 44 isolates) are clone types within patients that have been sampled less than or equal to two time points or where we do not have isolates sampled for more than 0.5 years. These have been removed because we are interested in the development/adaptation and evolution of phenotypes over the course of colonisation and infection.

Growth rate in artificial sputum medium (ASM)

The raw data

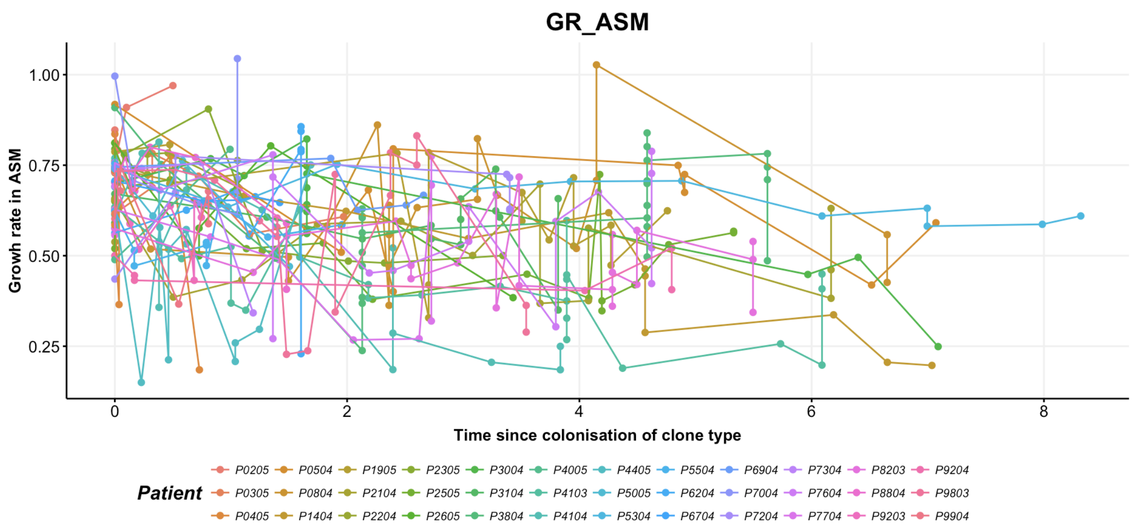

The one-to-one models

We start out comparing the growth rate in ASM (GR_ASM) one-to-one with all the remaining phenotypes. We always include the time since start of colonisation by the specific clone type (IageCT, or "ColT"" in the paper) to correct for the time factor, and the random effects of the patients using random smooths of time. Below you can find the specific code to generate the models

HIDE

```
#IageCT, Time since colonization
gammGRASM_time <- gam(GR_ASM ~ s(IageCT) + s(IageCT, Patient, bs = "fs", m = 1), method = "REML", data = phenoDF)

#GR_LB, Growth rate in LB
gammGRASM_LB <- gam(GR_ASM ~ s(IageCT) + s(GR_LB) + s(IageCT, Patient, bs = "fs", m = 1), method = "REML", data =
phenoDF)

#AztN, Aztreonam susceptibility
gammGRASM_azt <- gam(GR_ASM ~ s(IageCT) + s(log2(aztN)) + s(IageCT, Patient, bs = "fs", m = 1), method = "REML",
data = phenoDF)

#CipN, Ciprofloxacin susceptibility
gammGRASM_cip <- gam(GR_ASM ~ s(IageCT) + s(log2(cipN)) + s(IageCT, Patient, bs = "fs", m = 1), method = "REML",
data = phenoDF)

#AdhesionN, Adhesion to peglids
gammGRASM_adh <- gam(GR_ASM ~ s(IageCT) + s(log2(AdhesionN)) + s(IageCT, Patient, bs = "fs", m = 1), method = "RE
ML", data = phenoDF)

#aggASMavg, Estimated aggregation in ASM
gammGRASM_agg <- gam(GR_ASM ~ s(IageCT) + s(log2(ASMaggN)) + s(IageCT, Patient, bs = "fs", m = 1), method = "REML
", data = phenoDF)

#Protease, Protease production (1) or no protease production (0)
gammGRASM_prot <- gam(GR_ASM ~ s(IageCT) + Protease + s(IageCT, Patient, bs = "fs", m = 1), method = "REML", data
= phenoDF)

#Mucoid, Mucoid (1) or non-mucoid (0)
gammGRASM_muc <- gam(GR_ASM ~ s(IageCT) + Mucoid + s(IageCT, Patient, bs = "fs", m = 1), method = "REML", data =
phenoDF)

#hypermutator, hypermutator (1) or normomutator (0)
gammGRASM_mut <- gam(GR_ASM ~ s(IageCT) + hypermutator + s(IageCT, Patient, bs = "fs", m = 1), method = "REML", d
ata = phenoDF)
```

Diagnostic plots, examples

HIDE

```
par(mfrow=c(1,3), cex=1.1)
qq.gam(gammGRASM_time, pch=21, main = "QQ-plot")

h <- hist(residuals(gammGRASM_time), breaks="FD", xlab="Residuals", main="Histogram of residuals", col="lightgray
")
xfit <- seq(min(residuals(gammGRASM_time)), max(residuals(gammGRASM_time)), length = 40)
yfit <- dnorm(xfit, mean = mean(residuals(gammGRASM_time)), sd = sd(residuals(gammGRASM_time)))
yfit <- yfit * diff(h$mids[1:2]) * length(residuals(gammGRASM_time))
lines(xfit, yfit, col = "black", lwd = 2)

plot(fitted(gammGRASM_time), residuals(gammGRASM_time),
 xlab="Predicted scores", ylab="Residuals", main="Residuals plots")
```

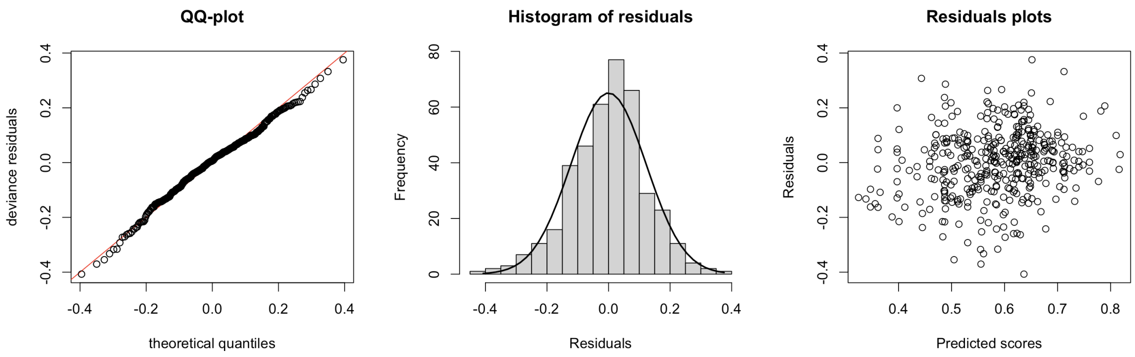

For the qq-plot: The black circles should fall on the red line, and this looks fine.

For the histogram of residuals: If the bars follow the bell-shaped line the residuals are normally distributed as they should be. This looks ok.

For the Residuals plot: The residuals vs. the predicted scores should have no pattern and this is true.

Model summaries

HIDE

```
summary(gammGRASM_time)
```

```
##
## Family: gaussian
## Link function: identity
##
## Formula:
## GR_ASM ~ s(IageCT) + s(IageCT, Patient, bs = "fs", m = 1)
##
## Parametric coefficients:
##             Estimate Std. Error t value Pr(>|t|)
## (Intercept)  0.58870    0.01521   38.72   <2e-16 ***
## ---
## Signif. codes:  0 '***' 0.001 '**' 0.01 '*' 0.05 '.' 0.1 ' ' 1
##
## Approximate significance of smooth terms:
##                         edf  Ref.df     F  p-value
## s(IageCT)             3.246   3.899 9.093 5.83e-07 ***
## s(IageCT,Patient) 47.014 232.000 0.734  < 2e-16 ***
## ---
## Signif. codes:  0 '***' 0.001 '**' 0.01 '*' 0.05 '.' 0.1 ' ' 1
##
## R-sq.(adj) =  0.381   Deviance explained = 45.9%
## -REML = -195.49  Scale est. = 0.017117   n = 399
```

IageCT has a significant impact on GR_ASM.

HIDE

```
par(mfrow=c(1,2), cex=1.1)
plot(gammGRASM_time,residuals=T,shade=T, main = "GRASM_time")
```

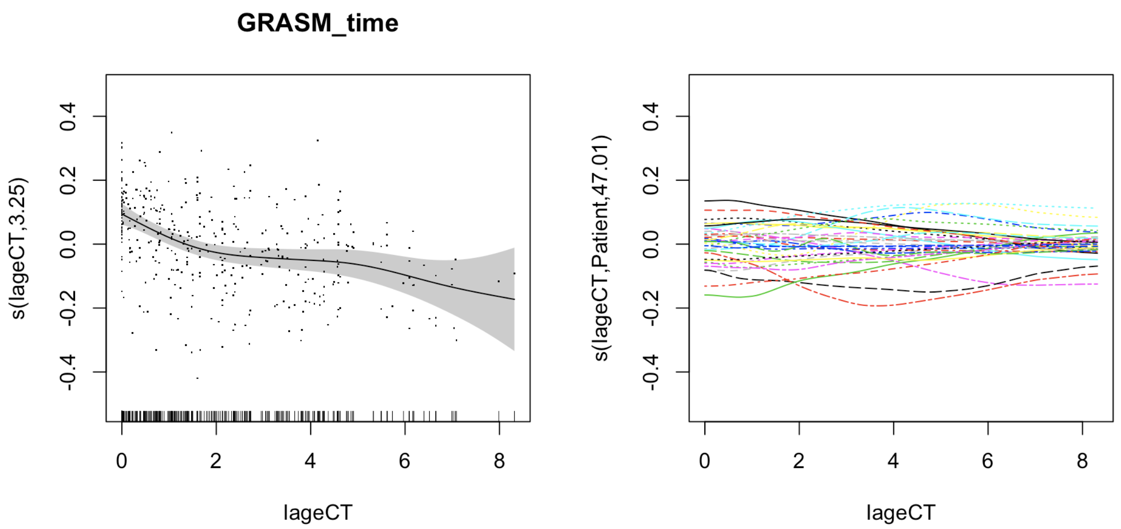

Models with multiple explanatory variables

The following model was created utilising the explanatory variables that we found to have a significant impact on the predictor in the one-on-one models (see the table of p-values at the top of the document).

HIDE

```
gammGRASM <- gam(GR_ASM ~ s(IageCT) + s(GR_LB) + s(log2(AdhesionN)) + s(log2(ASMaggN)) + s(IageCT, Patient, bs =
"fs", m = 1), method = "REML", data = phenoDF)
gammGRASM.1 <- gam(GR_ASM ~ s(IageCT) + s(GR_LB) + s(log2(ASMaggN)) + s(IageCT, Patient, bs = "fs", m = 1), metho
d = "REML", data = phenoDF)
```

Diagnostic plots

Diagnostic plots used to see if the model assumptions are upheld.

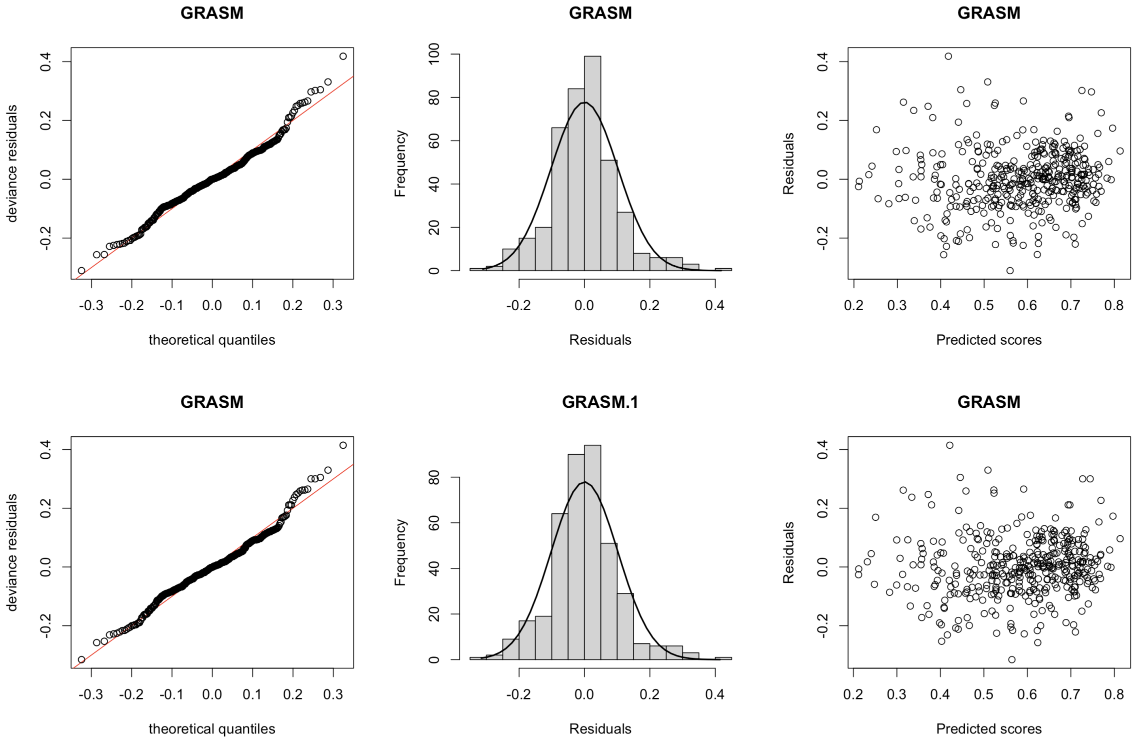

The better model

HIDE

```
compareML(gammGRASM.1,gammGRASM)
```

```
## gammGRASM.1: GR_ASM ~ s(IageCT) + s(GR_LB) + s(log2(ASMaggN)) + s(IageCT,
##     Patient, bs = "fs", m = 1)
##
## gammGRASM: GR_ASM ~ s(IageCT) + s(GR_LB) + s(log2(AdhesionN)) + s(log2(ASMaggN)) +
##     s(IageCT, Patient, bs = "fs", m = 1)
##
## Model gammGRASM.1 preferred: lower REML score (3.933), and lower df (2.000).
## -----
##         Model     Score Edf Difference     Df
## 1   gammGRASM -279.5097  11
## 2 gammGRASM.1 -283.4422   9      3.933 -2.000
##
## AIC difference: -2.48, model gammGRASM.1 has lower AIC.
```

```
## Warning in compareML(gammGRASM.1, gammGRASM): Only small difference in REML...
```

The reduced model is the preffered model, though they are not significantly different from each other.

Model summary

HIDE

```
summary(gammGRASM.1)
```

```
##
## Family: gaussian
## Link function: identity
##
## Formula:
## GR_ASM ~ s(IageCT) + s(GR_LB) + s(log2(ASMaggN)) + s(IageCT,
##     Patient, bs = "fs", m = 1)
##
## Parametric coefficients:
##             Estimate Std. Error t value Pr(>|t|)
## (Intercept) 0.582214   0.008179   71.18   <2e-16 ***
## ---
## Signif. codes:  0 '***' 0.001 '**' 0.01 '*' 0.05 '.' 0.1 ' ' 1
##
## Approximate significance of smooth terms:
##                        edf  Ref.df      F  p-value
## s(IageCT)            3.612   4.387  3.570 0.006947 **
## s(GR_LB)             3.709   4.576 41.399  < 2e-16 ***
## s(log2(ASMaggN))     2.969   3.715 22.025 3.63e-15 ***
## s(IageCT,Patient) 26.990 232.000  0.202 0.000797 ***
## ---
## Signif. codes:  0 '***' 0.001 '**' 0.01 '*' 0.05 '.' 0.1 ' ' 1
##
## R-sq.(adj) =  0.584   Deviance explained = 62.3%
## -REML = -283.44  Scale est. = 0.011509  n = 399
```

Growth rate in LB (GR_LB)

The raw data

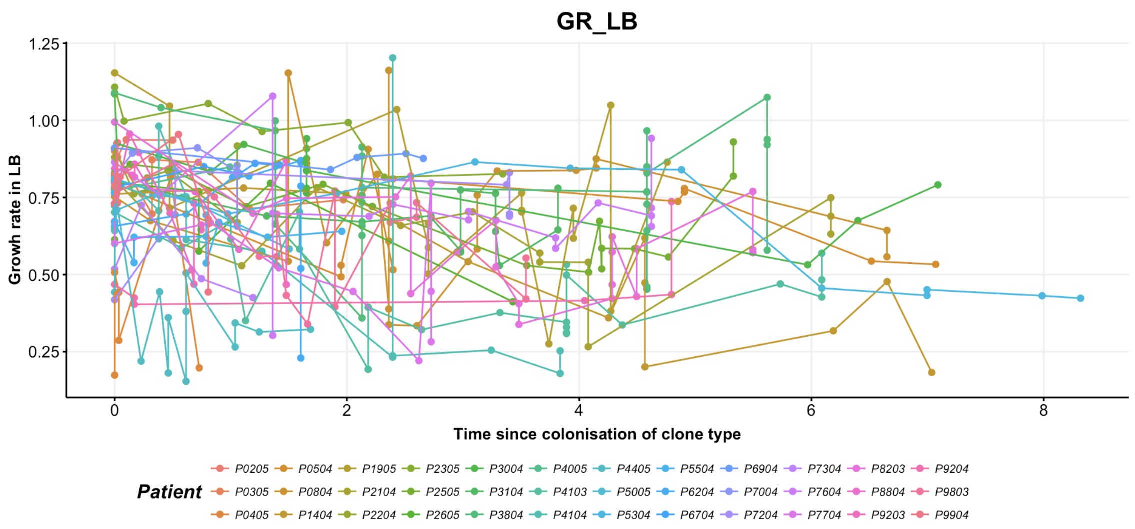

Models with multiple explanatory variables

The following model was created utilising the explanatory variables that we found to have a p-value of <0.05 for the impact on the predictor in the one-on-one models above.

HIDE

```
gammGRLB <- gam(GR_LB ~ s(IageCT) + s(GR_ASM) + s(log2(aztN)) + s(IageCT, Patient, bs = "fs", m = 1), method = "R
EML", data = phenoDF)
```

```
## Warning in gam.side(sm, X, tol = .Machine$double.eps^0.5): model has
## repeated 1-d smooths of same variable.
```

HIDE

```
gammGRLB.1 <- gam(GR_LB ~ s(IageCT) + s(GR_ASM) + s(IageCT, Patient, bs = "fs", m = 1), method = "REML", data = p
henoDF)
```

```
## Warning in gam.side(sm, X, tol = .Machine$double.eps^0.5): model has
## repeated 1-d smooths of same variable.
```

Diagnostic plots

Diagnostic plots used to see if the model assumptions are upheld.

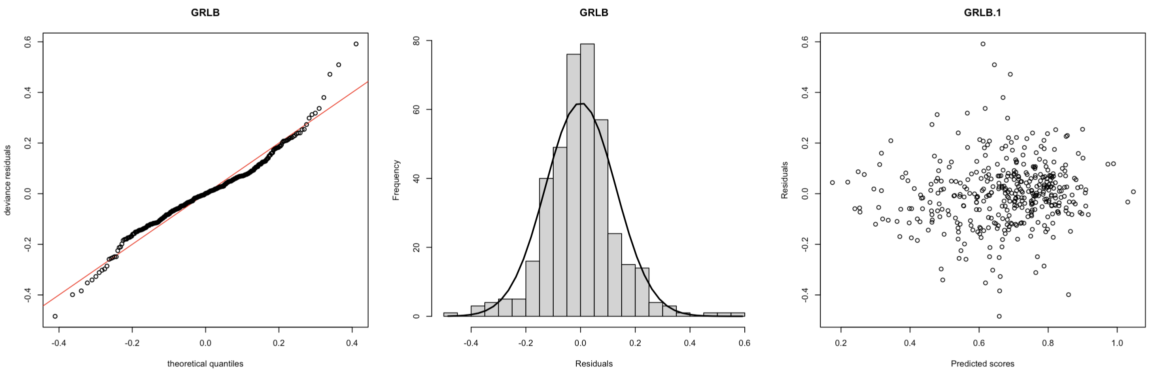

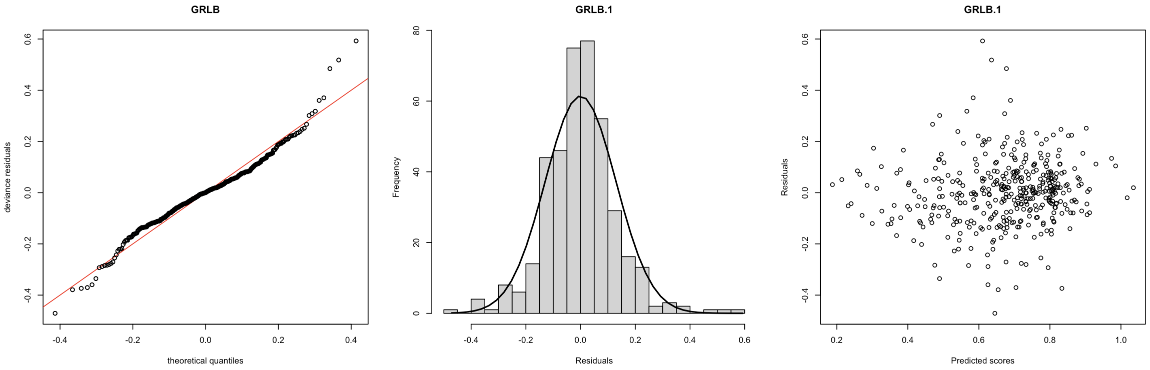

The better model

HIDE

```
compareML(gammGRLB.1,gammGRLB)
```

```
## gammGRLB.1: GR_LB ~ s(IageCT) + s(GR_ASM) + s(IageCT, Patient, bs = "fs",
##     m = 1)
##
## gammGRLB: GR_LB ~ s(IageCT) + s(GR_ASM) + s(log2(aztN)) + s(IageCT, Patient,
##     bs = "fs", m = 1)
##
## Model gammGRLB.1 preferred: lower REML score (2.120), and lower df (2.000).
## -----
##        Model     Score Edf Difference     Df
## 1   gammGRLB -176.6241   9
## 2 gammGRLB.1 -178.7445   7      2.120 -2.000
##
## AIC difference: 1.75, model gammGRLB has lower AIC.
```

```
## Warning in compareML(gammGRLB.1, gammGRLB): Only small difference in REML...
```

The reduced model is the better model, though they are not significantly different from each other.

Model summaries

HIDE

```
summary(gammGRLB.1)
```

```
##
## Family: gaussian
## Link function: identity
##
## Formula:
## GR_LB ~ s(IageCT) + s(GR_ASM) + s(IageCT, Patient, bs = "fs",
##     m = 1)
##
## Parametric coefficients:
##             Estimate Std. Error t value Pr(>|t|)
## (Intercept)  0.66762    0.01718   38.87   <2e-16 ***
## ---
## Signif. codes:  0 '***' 0.001 '**' 0.01 '*' 0.05 '.' 0.1 ' ' 1
##
## Approximate significance of smooth terms:
##                      edf  Ref.df      F  p-value
## s(IageCT)          4.403   5.364  4.145 0.000748 ***
## s(GR_ASM)          2.592   3.264 38.875  < 2e-16 ***
## s(IageCT,Patient) 34.027 230.000  0.625  < 2e-16 ***
## ---
## Signif. codes:  0 '***' 0.001 '**' 0.01 '*' 0.05 '.' 0.1 ' ' 1
##
## R-sq.(adj) =  0.561   Deviance explained = 60.6%
## -REML = -178.74  Scale est. = 0.018719  n = 399
```

Aztreonam sensitivity (aztN)

The raw data

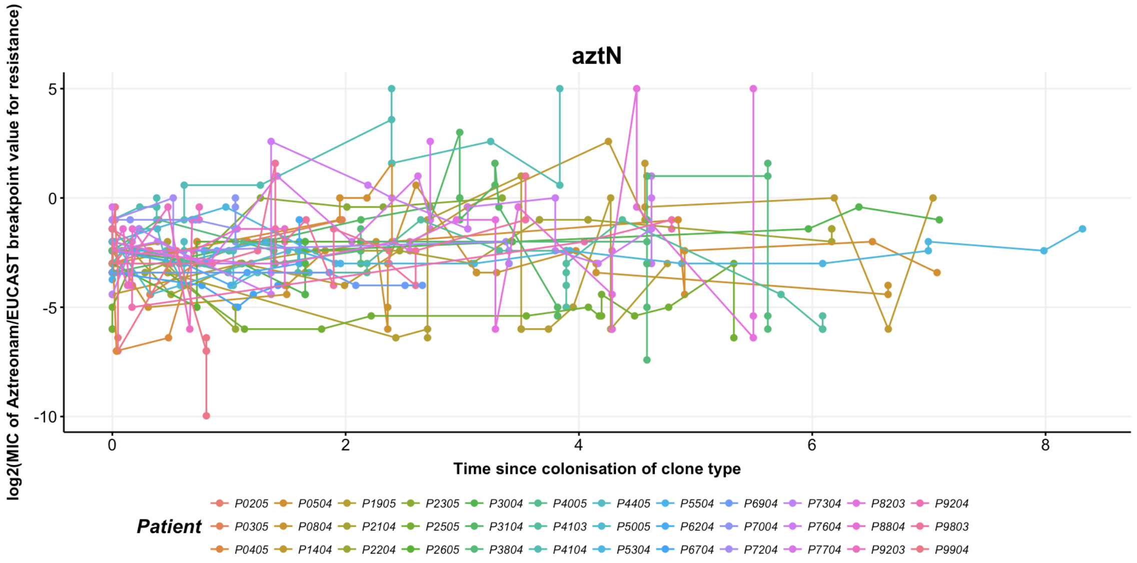

Models with multiple explanatory variables

The following model was created utilising the explanatory variables that we found to have a p-value of <0.05 for the impact on the predictor in the one-on-one models above.

HIDE

```
gammaztN <- gam(log2(aztN) ~ s(log2(AdhesionN)) + hypermutator + s(IageCT, Patient, bs = "fs", m = 1), method = "
REML", data = phenoDF)
```

Diagnostic plots

Diagnostic plots used to see if the model assumptions are upheld.

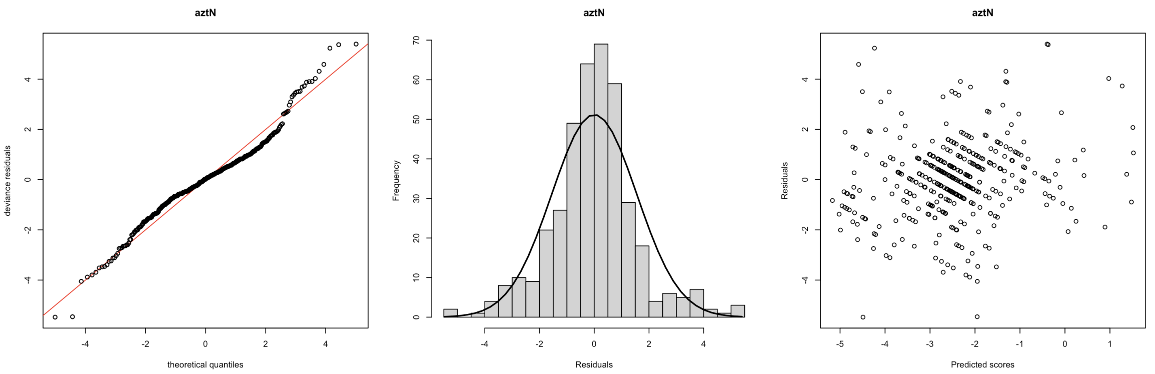

Model summary

HIDE

```
summary(gammaztN)
```

```
##
## Family: gaussian
## Link function: identity
##
## Formula:
## log2(aztN) ~ s(log2(AdhesionN)) + hypermutator + s(IageCT, Patient,
##     bs = "fs", m = 1)
##
## Parametric coefficients:
##               Estimate Std. Error t value Pr(>|t|)
## (Intercept)    -2.5388     0.2058 -12.338  < 2e-16 ***
## hypermutator1   2.0670     0.5686   3.635 0.000319 ***
## ---
## Signif. codes:  0 '***' 0.001 '**' 0.01 '*' 0.05 '.' 0.1 ' ' 1
##
## Approximate significance of smooth terms:
##                        edf  Ref.df     F p-value
## s(log2(AdhesionN))   1.003   1.005 6.692    0.01 *
## s(IageCT,Patient)   45.742 232.000 0.938  <2e-16 ***
## ---
## Signif. codes:  0 '***' 0.001 '**' 0.01 '*' 0.05 '.' 0.1 ' ' 1
##
## R-sq.(adj) =   0.38   Deviance explained = 45.5%
## -REML = 809.81  Scale est. = 2.7489    n = 399
```

Ciprofloxacin sensitivity (cipN)

The raw data

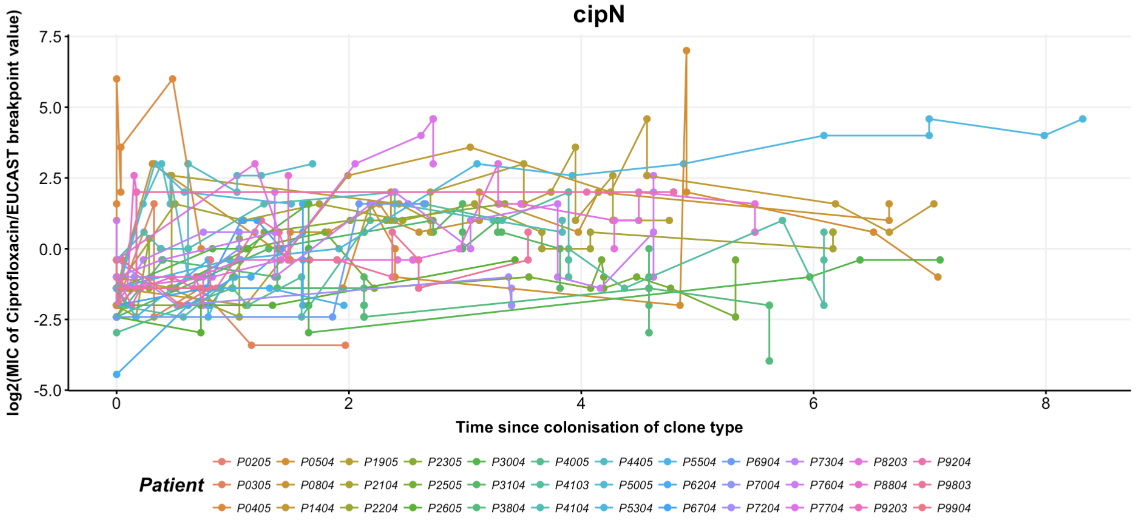

Models with multiple explanatory variables

The following model was created utilising the explanatory variables that we found to have a p-value of <0.05 for the impact on the predictor in the one-on-one models above.

HIDE

```
gammcipN <- gam(log2(cipN) ~ s(IageCT) + hypermutator + s(IageCT, Patient, bs = "fs", m = 1), method = "REML", da
ta = phenoDF)
```

```
## Warning in gam.side(sm, X, tol = .Machine$double.eps^0.5): model has
## repeated 1-d smooths of same variable.
```

Diagnostic plots

Diagnostic plots used to see if the model assumptions are upheld.

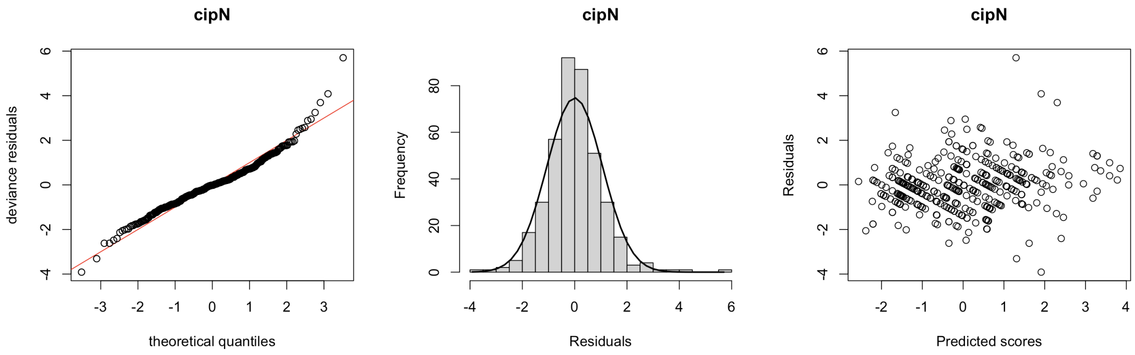

Model summary

HIDE

```
summary(gammcipN_mut)
```

```
##
## Family: gaussian
## Link function: identity
##
## Formula:
## log2(cipN) ~ s(IageCT) + hypermutator + s(IageCT, Patient, bs = "fs",
##     m = 1)
##
## Parametric coefficients:
##               Estimate Std. Error t value Pr(>|t|)
## (Intercept)   -0.02862    0.19680  -0.145   0.8844
## hypermutator1  1.05847    0.43988   2.406   0.0167 *
## ---
## Signif. codes:  0 '***' 0.001 '**' 0.01 '*' 0.05 '.' 0.1 ' ' 1
##
## Approximate significance of smooth terms:
##                       edf  Ref.df      F p-value
## s(IageCT)           3.598   4.278 11.449 5.5e-09 ***
## s(IageCT,Patient) 60.271 232.000  1.637 < 2e-16 ***
## ---
## Signif. codes:  0 '***' 0.001 '**' 0.01 '*' 0.05 '.' 0.1 ' ' 1
##
## R-sq.(adj) =  0.587   Deviance explained = 65.4%
## -REML = 692.57  Scale est. = 1.3531    n = 399
```

Adhesion to peglid (AdhesionN)

The raw data

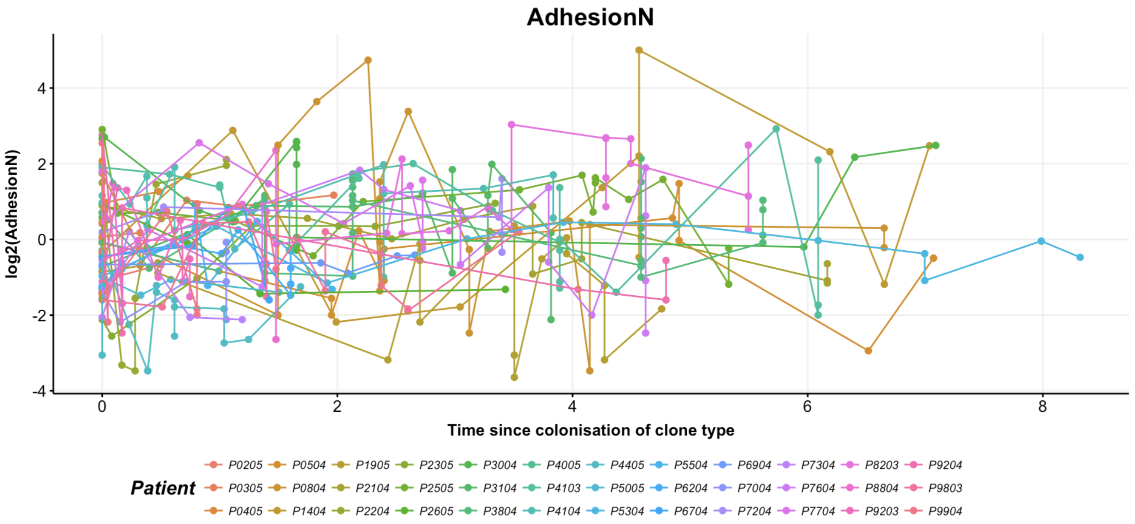

Models with multiple explanatory variables

The following model was created utilising the explanatory variables that we found to have a p-value of <0.05 for the impact on the predictor in the one-on-one models above.

HIDE

```
gammadh <- gam(log2(AdhesionN) ~ s(GR_ASM) + s(GR_LB) + s(log2(aztN)) + s(log2(ASMaggN)) + s(IageCT, Patient, bs = "fs", m = 1), method = "REML", data = phenoDF)

gammadh.1 <- gam(log2(AdhesionN) ~ s(GR_LB) + s(log2(aztN)) + s(log2(ASMaggN)) + s(IageCT, Patient, bs = "fs", m = 1), method = "REML", data = phenoDF)
```

Diagnostic plots

Diagnostic plots used to see if the model assumptions are upheld.

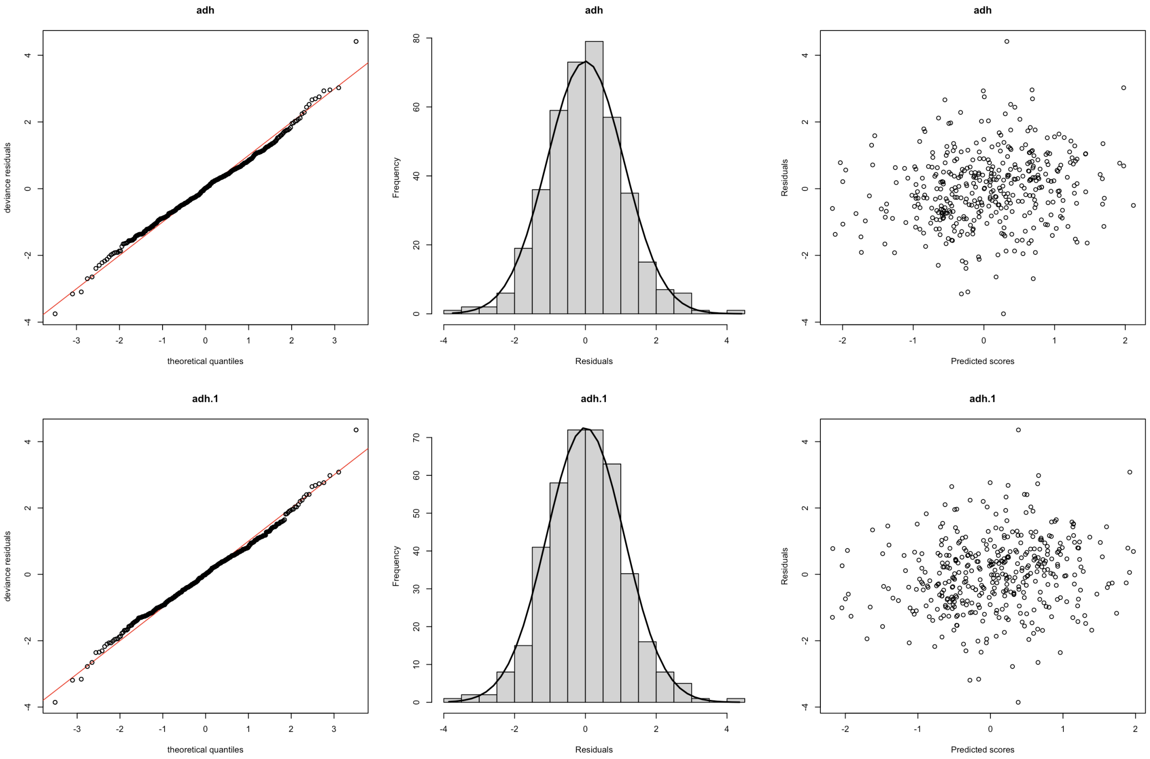

The better model

HIDE

```
compareML(gammadh,gammadh.1)
```

```
## gammadh: log2(AdhesionN) ~ s(GR_ASM) + s(GR_LB) + s(log2(aztN)) + s(log2(ASMaggN)) +
##     s(IageCT, Patient, bs = "fs", m = 1)
##
## gammadh.1: log2(AdhesionN) ~ s(GR_LB) + s(log2(aztN)) + s(log2(ASMaggN)) +
##     s(IageCT, Patient, bs = "fs", m = 1)
##
## Model gammadh.1 preferred: lower REML score (0.428), and lower df (2.000).
## -----
##       Model    Score Edf Difference    Df
## 1   gammadh 668.7578  11
## 2 gammadh.1 668.3301   9     -0.428 2.000
##
## AIC difference: 0.70, model gammadh.1 has lower AIC.
```

```
## Warning in compareML(gammadh, gammadh.1): Only small difference in REML...
```

gammadh.1 seems to be the better model, though the models are not significantly different from each other.

Model summary

HIDE

```
summary(gammadh.1)
```

```
##
## Family: gaussian
## Link function: identity
##
## Formula:
## log2(AdhesionN) ~ s(GR_LB) + s(log2(aztN)) + s(log2(ASMaggN)) +
##     s(IageCT, Patient, bs = "fs", m = 1)
##
## Parametric coefficients:
##             Estimate Std. Error t value Pr(>|t|)
## (Intercept) -0.01225    0.12789  -0.096    0.924
##
## Approximate significance of smooth terms:
##                      edf  Ref.df     F  p-value
## s(GR_LB)           4.722   5.754 2.850 0.012931 *
## s(log2(aztN))      1.002   1.003 6.537 0.010916 *
## s(log2(ASMaggN))   2.005   2.526 8.182 0.000149 ***
## s(IageCT,Patient) 36.814 231.000 0.611  < 2e-16 ***
## ---
## Signif. codes:  0 '***' 0.001 '**' 0.01 '*' 0.05 '.' 0.1 ' ' 1
##
## R-sq.(adj) =   0.35   Deviance explained = 42.3%
## -REML = 668.33  Scale est. = 1.3523    n = 399
```

Estimated aggregation in ASM (aggASMavg)

The raw data

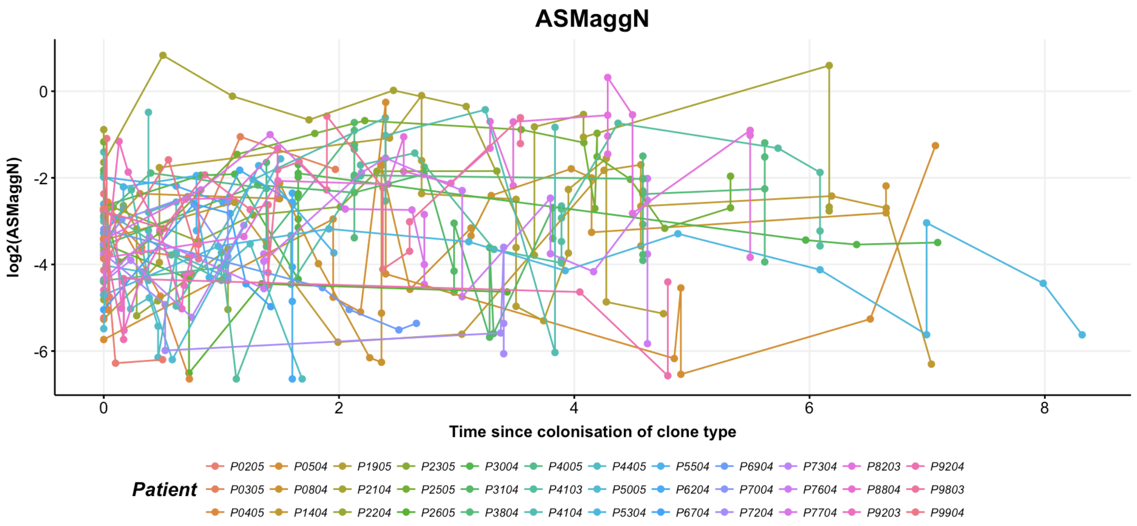

Models with multiple explanatory variables

The following model was created utilising the explanatory variables that we found to have a p-value of <0.05 for the impact on the predictor in the one-on-one models above.

HIDE

```
gammagg <- gam(log2(ASMaggN) ~ s(GR_ASM) + s(log2(AdhesionN)) + Protease + s(IageCT, Patient, bs = "fs", m = 1),
method = "REML", data = phenoDF)
```

Diagnostic plots

Diagnostic plots used to see if the model assumptions are upheld.

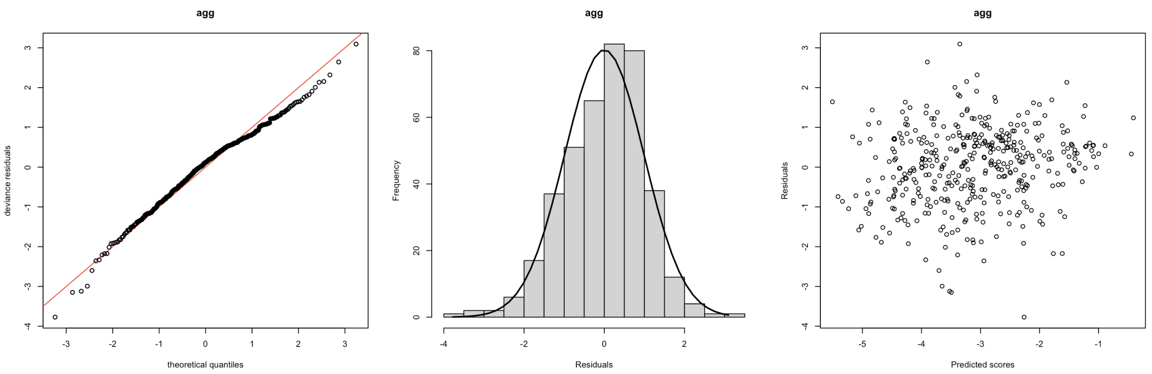

Model summaries

HIDE

```
summary(gammagg)
```

```
##
## Family: gaussian
## Link function: identity
##
## Formula:
## log2(ASMaggN) ~ s(GR_ASM) + s(log2(AdhesionN)) + Protease + s(IageCT,
##     Patient, bs = "fs", m = 1)
##
## Parametric coefficients:
##             Estimate Std. Error t value Pr(>|t|)
## (Intercept)  -4.2625     0.2629 -16.212  < 2e-16 ***
## Protease1     1.0155     0.2357   4.308 2.16e-05 ***
## ---
## Signif. codes:  0 '***' 0.001 '**' 0.01 '*' 0.05 '.' 0.1 ' ' 1
##
## Approximate significance of smooth terms:
##                        edf  Ref.df      F  p-value
## s(GR_ASM)            3.601   4.465 10.852 1.25e-08 ***
## s(log2(AdhesionN))   3.340   4.184  3.876  0.00384 **
## s(IageCT,Patient)   49.676 232.000  0.974  < 2e-16 ***
## ---
## Signif. codes:  0 '***' 0.001 '**' 0.01 '*' 0.05 '.' 0.1 ' ' 1
##
## R-sq.(adj) =  0.502   Deviance explained = 57.4%
## -REML =  649.5  Scale est. = 1.149     n = 399
```

Mutation accumulation analyses

The data

For the analysis of the accumulation of mutation over time, we have added filtering step where mutations present in all isolates of a lineage (a clone type within a specific patient) were removed from the analysis. This was done to identify mutations that had occurred within the time the specific clone type had been observed and recorded in the patient. The raw data of mutations that have accumulated have previously been published in Marvig *et al.*: "Convergent evolution and adaptation of *Pseudomonas aeruginosa* within patients with cystic fibrosis", Nature Genetics **47**, (2015).

```
## # A tibble: 5 x 7
##    ID_1 Patient Genotype IageCT nonsynSNPs INDELs allmutsPT
##   <chr>  <fctr>   <fctr>  <dbl>      <int>  <int>     <int>
## 1     1   P6204     DK03  0.000          3      7        12
## 2     2   P6204     DK03  0.616          1      5         7
## 3     3   P6204     DK03  1.156          3      8        13
## 4     4   P6204     DK03  1.016          1     11        15
## 5     6   P2505     DK04  0.000          7      7        15
```

Non-synonymous SNPs, indels, and all mutations

The raw data

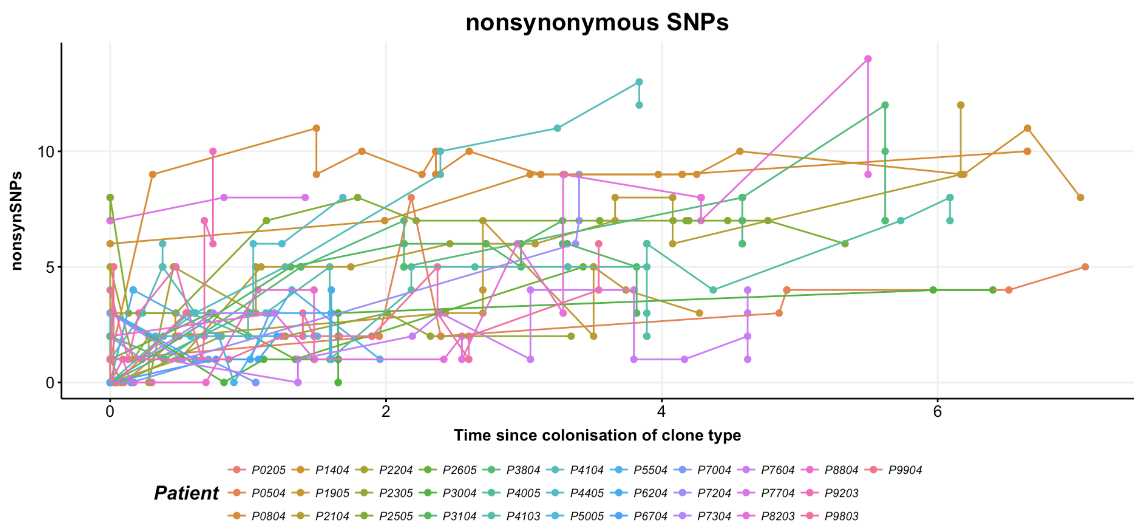

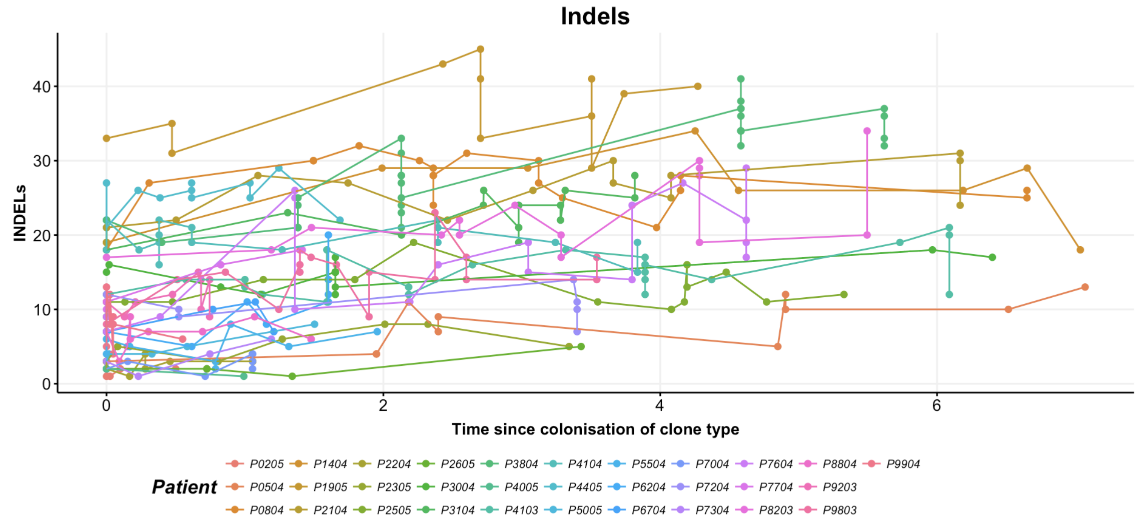

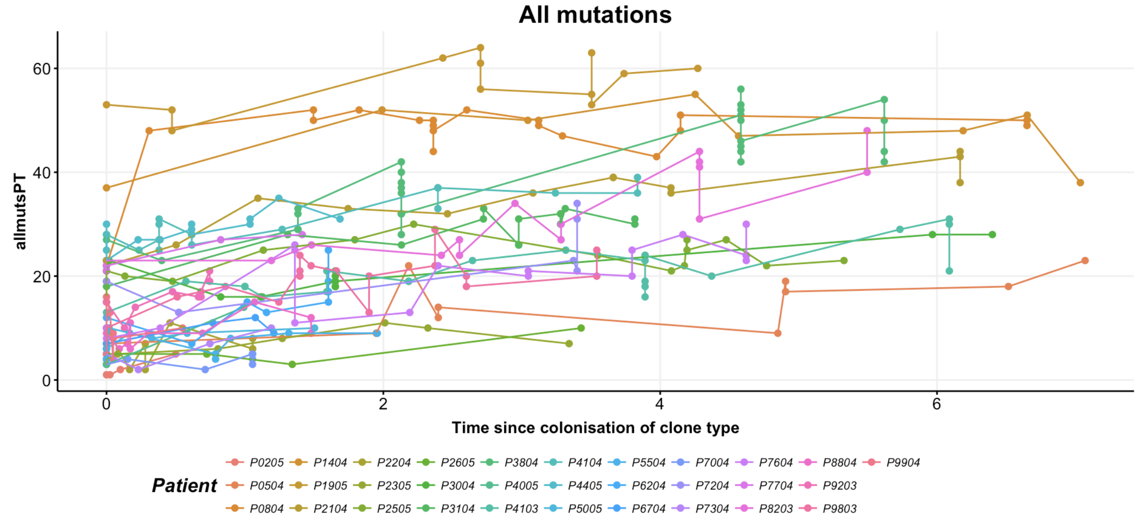

The models

We are aware that the mutations are counts, but because we have so big a spread of numbers we have stayed with the default distribution first, as if they were continuous data.

HIDE

```
#nonsynSNPs
gammPT_nonsynSNPs <- gam(nonsynSNPs ~ s(IageCT) + s(IageCT, Patient, bs = "fs", m = 1), method = "REML", data = M
Mpt)

#INDELs
gammPT_INDELs <- gam(INDELs ~ s(IageCT) + s(IageCT, Patient, bs = "fs", m = 1), method = "REML", data = MMpt)

#allmutsPT
gammPT_allmutsPT <- gam(allmutsPT ~ s(IageCT) + s(IageCT, Patient, bs = "fs", m = 1), method = "REML", data = MMp
t)
```

Diagnostic plots

Diagnostic plots as the examples shown above was used to see if the model assumptions were upheld, and all plots are reasonable.

Model summaries

HIDE

```
summary(gammPT_nonsynSNPs)
```

```
##
## Family: gaussian
## Link function: identity
##
## Formula:
## nonsynSNPs ~ s(IageCT) + s(IageCT, Patient, bs = "fs", m = 1)
##
## Parametric coefficients:
##             Estimate Std. Error t value Pr(>|t|)
## (Intercept)   4.0876     0.3601   11.35   <2e-16 ***
## ---
## Signif. codes:  0 '***' 0.001 '**' 0.01 '*' 0.05 '.' 0.1 ' ' 1
##
## Approximate significance of smooth terms:
##                      edf  Ref.df      F p-value
## s(IageCT)          1.919   2.173 32.648   7e-14 ***
## s(IageCT,Patient) 67.085 197.000  3.829  <2e-16 ***
## ---
## Signif. codes:  0 '***' 0.001 '**' 0.01 '*' 0.05 '.' 0.1 ' ' 1
##
## R-sq.(adj) =  0.802   Deviance explained = 84.2%
## -REML = 673.81  Scale est. = 1.9847    n = 338
```

HIDE

```
summary(gammPT_INDELs)
```

```
##
## Family: gaussian
## Link function: identity
##
## Formula:
## INDELs ~ s(IageCT) + s(IageCT, Patient, bs = "fs", m = 1)
##
## Parametric coefficients:
##             Estimate Std. Error t value Pr(>|t|)
## (Intercept)   14.026      1.533    9.15   <2e-16 ***
## ---
## Signif. codes:  0 '***' 0.001 '**' 0.01 '*' 0.05 '.' 0.1 ' ' 1
##
## Approximate significance of smooth terms:
##                      edf  Ref.df     F  p-value
## s(IageCT)          2.656   3.096 11.11 4.14e-07 ***
## s(IageCT,Patient) 60.271 196.000 12.59  < 2e-16 ***
## ---
## Signif. codes:  0 '***' 0.001 '**' 0.01 '*' 0.05 '.' 0.1 ' ' 1
##
## R-sq.(adj) =  0.913   Deviance explained = 92.9%
## -REML = 935.57  Scale est. = 8.7037    n = 338
```

HIDE

```
summary(gammPT_allmutsPT)
```

```
##
## Family: gaussian
## Link function: identity
##
## Formula:
## allmutsPT ~ s(IageCT) + s(IageCT, Patient, bs = "fs", m = 1)
##
## Parametric coefficients:
##             Estimate Std. Error t value Pr(>|t|)
## (Intercept)   21.353      2.172   9.832   <2e-16 ***
## ---
## Signif. codes:  0 '***' 0.001 '**' 0.01 '*' 0.05 '.' 0.1 ' ' 1
##
## Approximate significance of smooth terms:
##                       edf  Ref.df     F p-value
## s(IageCT)           2.722   3.149 25.84 1.6e-15 ***
## s(IageCT,Patient) 65.057 197.000 18.59 < 2e-16 ***
## ---
## Signif. codes:  0 '***' 0.001 '**' 0.01 '*' 0.05 '.' 0.1 ' ' 1
##
## R-sq.(adj) =  0.944   Deviance explained = 95.6%
## -REML = 1002.2  Scale est. = 12.182    n = 338
```

Mutation of *gyrA*-*gyrB*

The data

From the molecular analysis previously done in Marvig et al.: "Convergent evolution and adaptation of Pseudomonas aeruginosa within patients with cystic fibrosis", Nature Genetics 47, (2015), we have extracted information of mutations in the genes: *gyrA* and *gyrB*. This is shown as presence/absence (1/0) of any nonsynonymous mutation (that is, missense SNPs, nonsense SNPs, insertions and deletions (indels)) for each of the isolates sequenced.

```
## # A tibble: 6 x 6
##                  ID Genotype IageCT    GR_ASM   gyrA   gyrB
##               <chr>   <fctr>  <dbl>     <dbl> <fctr> <fctr>
## 1 C04P2505I111511b     DK04  4.194 0.3754161      0      0
## 2 C04P2505I100411a     DK04  4.081 0.3828657      0      0
## 3 C04P2505I102308a     DK04  1.133 0.5194534      0      0
## 4 C04P2505I110811a     DK04  4.175 0.7243063      0      0
## 5 C06P4405I031611a     DK06  0.387 0.5781520      1      0
## 6 C06P4405I102710a     DK06  0.000 0.5769268      0      0
```

The models

Since we already established models with multiple explanatory phenotypic variables (see the top of the document), these are also used for the purpose of identifying mutational impacts on the different phenotypes of interest.

gyrA, *gyrB*, and *nfxB*

HIDE

```
gammcipN_gyr <- gam(log2(cipN) ~ s(IageCT) + hypermutator + gyrA + gyrB + nfxB + s(IageCT, Patient, bs = "fs", m
= 1), method = "REML", data = mut_pheno)

gammAdh_gyr <- gam(log2(AdhesionN) ~ s(GR_LB) + s(log2(aztN)) + s(log2(ASMaggN)) + gyrA + gyrB + nfxB + s(IageCT,
Patient, bs = "fs", m = 1), method = "REML", data = mut_pheno)
```

The diagnostic plots were visually inspected, and they suggest that the model assumptions are upheld.

Model summaries

```
##
## Family: gaussian
## Link function: identity
##
## Formula:
## log2(cipN) ~ s(IageCT) + hypermutator + gyrA + gyrB + nfxB +
##     s(IageCT, Patient, bs = "fs", m = 1)
##
## Parametric coefficients:
##               Estimate Std. Error t value Pr(>|t|)
## (Intercept)    -0.5706     0.1507  -3.787 0.000182 ***
## hypermutator1   0.7879     0.3965   1.987 0.047776 *
## gyrA1           1.1756     0.2892   4.064 6.08e-05 ***
## gyrB1           0.8506     0.2932   2.901 0.003977 **
## nfxB1           1.5997     0.2633   6.076 3.54e-09 ***
## ---
## Signif. codes:  0 '***' 0.001 '**' 0.01 '*' 0.05 '.' 0.1 ' ' 1
##
## Approximate significance of smooth terms:
##                     edf  Ref.df     F  p-value
## s(IageCT)          3.40   4.024 8.605 1.21e-06 ***
## s(IageCT,Patient) 47.99 209.000 1.018  < 2e-16 ***
## ---
## Signif. codes:  0 '***' 0.001 '**' 0.01 '*' 0.05 '.' 0.1 ' ' 1
##
## R-sq.(adj) =  0.612   Deviance explained =   67%
## -REML = 594.09  Scale est. = 1.0884    n = 373
```

```
##
## Family: gaussian
## Link function: identity
##
## Formula:
## log2(AdhesionN) ~ s(GR_LB) + s(log2(aztN)) + s(log2(ASMaggN)) +
##     gyrA + gyrB + nfxB + s(IageCT, Patient, bs = "fs", m = 1)
##
## Parametric coefficients:
##             Estimate Std. Error t value Pr(>|t|)
## (Intercept)  -0.1041     0.1317  -0.790    0.430
## gyrA1        -0.3578     0.2711  -1.320    0.188
## gyrB1         1.2983     0.2910   4.462 1.12e-05 ***
## nfxB1         0.2261     0.2676   0.845    0.399
## ---
## Signif. codes:  0 '***' 0.001 '**' 0.01 '*' 0.05 '.' 0.1 ' ' 1
##
## Approximate significance of smooth terms:
##                      edf  Ref.df     F  p-value
## s(GR_LB)           5.218   6.313 2.861 0.008027 **
## s(log2(aztN))      1.122   1.222 4.871 0.017482 *
## s(log2(ASMaggN))   2.195   2.765 6.066 0.000737 ***
## s(IageCT,Patient) 32.080 208.000 0.458 3.93e-10 ***
## ---
## Signif. codes:  0 '***' 0.001 '**' 0.01 '*' 0.05 '.' 0.1 ' ' 1
##
## R-sq.(adj) =  0.373   Deviance explained = 44.7%
## -REML = 619.01  Scale est. = 1.3191    n = 373
```

Supplementary References

1. Eugster, M. J. A. & Leisch, F. (2009). From Spider-Man to hero: Archetypal analysis in R. J. Stat. Softw. 30, 1–23. https://doi.org/10.18637/jss.v030.i08
2. Eugster, M. J. A., & Leisch, F. (2011). Weighted and robust archetypal analysis. Computational Statistics and Data Analysis, 55(3), 1215–1225. https://doi.org/10.1016/j.csda.2010.10.017
3. Thøgersen, J. C., Mørup, M., Damkiær, S., Molin, S., & Jelsbak, L. (2013). Archetypal analysis of diverse Pseudomonas aeruginosa transcriptomes reveals adaptation in cystic fibrosis airways. BMC Bioinformatics, 14, 279. https://doi.org/10.1186/1471-2105-14-279
4. Fernandez, M., Wilson, H., & Barnard, A. S. (2017). Impact of Distributions on the Archetypes and Prototypes in Heterogenous Nanoparticle Ensembles. Nanoscale, 9, 832–843. https://doi.org/10.1039/C6NR07102C
5. Wood, S. N. Generalized additive models: an introduction with R. (2nd Ed.) CRC Press, 2006. ISBN: 9781315370279.
6. Wood, S. N., Pya, N. & Säfken, B. Smoothing Parameter and Model Selection for General Smooth Models. J. Am. Stat. Assoc. 111, 1548–1563 (2016). https://doi.org/10.1080/01621459.2016.1180986
7. Wood, S.N. (2011) Fast stable restricted maximum likelihood and marginal likelihood estimation of semiparametric generalized linear models. Journal of the Royal Statistical Society (B) 73(1):3-36. https://doi.org/10.1111/j.1467-9868.2010.00749.x
8. Wood, S. N. (2013) On p-values for smooth components of an extended generalized additive model. Biometrika 100, 221–228. https://doi.org/10.1093/biomet/ass048
9. Hadley Wickham (2017). tidyverse: Easily Install and Load the 'Tidyverse'. R package version 1.2.1. https://CRAN.R-project.org /package=tidyverse
10. van Rij J, Wieling M, Baayen R and van Rijn H (2017). "itsadug: Interpreting Time Series and Autocorrelated Data Using GAMMs." R package version 2.3. https://CRAN.R-project.org /package=itsadug
11. Jeffrey B. Arnold (2017). ggthemes: Extra Themes, Scales and Geoms for 'ggplot2'. R package version 3.4.0. https://CRAN.R-project.org /package=ggthemes
12. Yihui Xie (2017). knitr: A General-Purpose Package for Dynamic Report Generation in R. R package version 1.17. https://CRAN.R-project.org /package=knitr
13. Hao Zhu (2017). kableExtra: Construct Complex Table with 'kable' and Pipe Syntax. R package version 0.6.1. https://CRAN.R-project.org /package=kableExtra
