# Supplementary material for: Evolutionary highways to persistent bacterial infection
Source: Nat Commun. 2019 Feb 7;10:629. doi: 10.1038/s41467-019-08504-7 (PMC6367392; doi:10.1038/s41467-019-08504-7)
Supplement: Supplementary file 3 — Description of Additional Supplementary Files [file 41467_2019_8504_MOESM3_ESM.pdf]

Description of Additional Supplementary Files

File Name: Supplementary Data 1

Description: Isolate collection. ID: The id of the isolate. SeqID: Sequence id of isolate for internal use and can be referenced to the isolate collection published and analyzed in Marvig et al.: "Convergent evolution and adaptation of Pseudomonas aeruginosa within patients with cystic fibrosis", Nature Genetics 47, (2015). SRA_accNo: Accession number for sequences published previously in Marvig et al.: "Convergent evolution and adaptation of Pseudomonas aeruginosa within patients with cystic fibrosis", Nature Genetics 47, (2015). Genotype: The clone type/genotype of the isolate. Patient: The patient wherefrom the isolate has been sampled. Date: Date of sampling, using American standard (month/day/year). IageCT: The "Infection age of the Clone Type", the time (in years) since the clone type of the specific isolate was first detected in the patient wherefrom the specific isolate was sampled. This is refered to in the study as "the colonization time". Origin: The origin of the isolate. azt: Minimum inhibitory concentration (MIC) of Aztreonam. aztN: Normalised MIC of Aztreonam. Normalised against the EUCAST breakpoint values, so that 1 and below is sensitive and above 1 equals resistant. cip: MIC of Ciprofloxacin. cipN: Normalised MIC of Ciprofloxacin. Normalised against the EUCAST breakpoint values, so that 1 and below is sensitive and above 1 equals resistant. AdhesionN: OD of crystal violet normalised against 20h of growth. GT_LB: Generation time in LB. GT_LB_SD: Standard deviation of generation time replicates in LB. GR_LB: Growth rate in LB, converted from average generation time in LB. GT_ASM: Generation time in ASM. GT_ASM_SD: Standard deviation of generation time replicates in ASM. Generation times of isolates with an NA only grew in one well of 4 replicates. GR_ASM: Growth rate in ASM, converted from average generation time in ASM. Protease: Protease production positive "1" or negative "0". Mucoid: Mucoid "1" or non-mucoid "0". aggASMavg: Average estimated aggregation measured in ASM. hypermutator: Hypermutator "1" or normo-mutator "0". singleton: singleton "1" not singleton "0", as defined in the materials and methods. ASMaggN: Average estimated aggregation measured in ASM, shifted away from zero with 0.01 (as is done in the archetype analysis) to be able to log transform in the GAMMs.

File Name: Supplementary Data 2

Description: dN/dS calculations based on SNPs previously called in Marvig et al. (2015) Nature Genetics. Probability of neutral selection is calculated using the function BINOM.DIST() in excel. We have included lineages with isolates older than 3 years and with more than two isolates being 0-3 years or 3-n years (n=total number of years collected from the given patient).
