# Supplementary material for: Evolutionary highways to persistent bacterial infection
Source: Nat Commun. 2019 Feb 7;10:629. doi: 10.1038/s41467-019-08504-7 (PMC6367392; doi:10.1038/s41467-019-08504-7)
Supplement: Supplementary file 6 — Reporting Summary [file 41467_2019_8504_MOESM6_ESM.pdf]

nature research

Corresponding author(s): Jennifer A Bartell
Lea M Sommer

Reporting Summary

Nature Research wishes to improve the reproducibility of the work that we publish. This form provides structure for consistency and transparency in reporting. For further information on Nature Research policies, see Authors & Referees and the Editorial Policy Checklist.

Statistical parameters

When statistical analyses are reported, confirm that the following items are present in the relevant location (e.g. figure legend, table legend, main text, or Methods section).

n/a	Confirmed	
☐	☒	The exact sample size (n) for each experimental group/condition, given as a discrete number and unit of measurement
☐	☒	An indication of whether measurements were taken from distinct samples or whether the same sample was measured repeatedly
☐	☒	The statistical test(s) used AND whether they are one- or two-sided<br>*Only common tests should be described solely by name; describe more complex techniques in the Methods section.*
☐	☒	A description of all covariates tested
☐	☒	A description of any assumptions or corrections, such as tests of normality and adjustment for multiple comparisons
☐	☒	A full description of the statistics including central tendency (e.g. means) or other basic estimates (e.g. regression coefficient) AND variation (e.g. standard deviation) or associated estimates of uncertainty (e.g. confidence intervals)
☐	☒	For null hypothesis testing, the test statistic (e.g. F, t, r) with confidence intervals, effect sizes, degrees of freedom and P value noted<br>*Give P values as exact values whenever suitable.*
☒	☐	For Bayesian analysis, information on the choice of priors and Markov chain Monte Carlo settings
☐	☒	For hierarchical and complex designs, identification of the appropriate level for tests and full reporting of outcomes
☐	☒	Estimates of effect sizes (e.g. Cohen's d, Pearson's r), indicating how they were calculated
☐	☒	Clearly defined error bars<br>*State explicitly what error bars represent (e.g. SD, SE, CI)*

Our web collection on statistics for biologists may be useful.

Software and code

Policy information about availability of computer code

Data collection	Growth rates were calculated using a sliding window approach via custom R code as described in the methods section of the manuscript. This code is available on request.
Data analysis	We provide R markdown documents that fully describe and enable replication of our data modeling using R 3.4.0 and R packages as described for archetype analysis and generalized additive mixed models.

For manuscripts utilizing custom algorithms or software that are central to the research but not yet described in published literature, software must be made available to editors/reviewers upon request. We strongly encourage code deposition in a community repository (e.g. GitHub). See the Nature Research guidelines for submitting code & software for further information.

Data

Policy information about availability of data

All manuscripts must include a data availability statement. This statement should provide the following information, where applicable:

- Accession codes, unique identifiers, or web links for publicly available datasets
- A list of figures that have associated raw data
- A description of any restrictions on data availability

We provide our complete phenotype dataset in raw form as a supplemental spreadsheet and include a visualization and summary statistics of normalized data in

Figure 2. Data normalization, processing and construction of all models was performed in R as described above and all essential code for reproduction of these steps is provided in R Markdown format in supplemental files 1-2. These files also include code for replicating the model visualizations of Figure 3A-D and Figure 4A-C,E. Code to reproduce various secondary analysis figures is available on request. All genomic information is publicly available as described in Marvig et al. 2015 and accession codes are included in Supplementary Data 1 for isolates included in genomic analyses.

Field-specific reporting

Please select the best fit for your research. If you are not sure, read the appropriate sections before making your selection.

☐ Life sciences ☐ Behavioural & social sciences ☒ Ecological, evolutionary & environmental sciences

For a reference copy of the document with all sections, see nature.com/authors/policies/ReportingSummary-flat.pdf

Ecological, evolutionary & environmental sciences study design

All studies must disclose on these points even when the disclosure is negative.

Study description	We phenotypically characterize clinical Pseudomonas aeruginosa isolates which have evolved in the lungs of young cystic fibrosis patients for up to 10 years by 8 traits assessed via high throughput screens. We use two data modeling approaches to map adaptation, identifying significant evolutionary trends that contribute to persistence of P. aeruginosa in our CF patient cohort.
Research sample	We study 443 clinical Pseudomonas aeruginosa isolates from a cohort of 39 children with CF (median age at first P. aeruginosa isolate = 8.1 years) treated at the Copenhagen CF Centre at Rigshospitalet and capture the early period of adaptation, spanning 0.2-10.2 years of colonization by a total of 52 clone types. Of these isolates, 373 were previously characterized in a molecular study of adaptation (Marvig et al., 2015).
Sampling strategy	Samples were collected every month in the outpatient cystic fibrosis clinic of Rigshospitalet in Copenhagen. If P. aeruginosa was cultured, it was stored at -80C. Due to economic considerations, representative samples were chosen from this larger collection to span the patient's infection. Samples always included the first and last isolate of the collection period for each patient and any isolate collected after an extended period of negative cultures for that patient. If a patient underwent sinus surgery, these isolates were also included to capture spatial structure of the infection. Patients were chosen based on age, infection status (P. aeruginosa positive), and in a subset of older youth, the absence of previous P. aeruginosa positive cultures to capture the early period of adaptation.
Data collection	Isolates were collected under the supervision of Dr.Med. Helle K Johansen, and phenotyping was performed by Janus A Haagensen.
Timing and spatial scale	Isolates were collected continuously between 2002 and 2014. Isolates were collected from both the upper and lower airways.
Data exclusions	We specifically excluded two patients due to their long-running concurrent infection with multiple clone types as complex outliers within our cohort, where clonal interactions complicated our modeling in comparison to the monoclonal infections of the remaining patients.
Reproducibility	For all phenotypes except the antibiotic MIC tests, phenotypic analysis was carried out by stabbing from a 96 well master plate pre-frozen with overnight cultures diluted with 50% glycerol at a ratio of 1:1 and four technical replicates were produced for each isolate. For MICs, the E-test was carried out multiple times for P. aeruginosa PAO1 to test reproducibility and ensure uniform readouts. All single E-tests were conducted by the same technician to avoid read bias.
Randomization	This is not relevant for the study. Isolates were associated with patients and no patients were divided into groups.
Blinding	Blinding was not relevant in our study.

Did the study involve field work? ☐ Yes ☒ No

Reporting for specific materials, systems and methods

Materials & experimental systems

n/a	Involved in the study
☐	☒ Unique biological materials
☒	☐ Antibodies
☒	☐ Eukaryotic cell lines
☒	☐ Palaeontology
☒	☐ Animals and other organisms
☒	☐ Human research participants

Methods

n/a	Involved in the study
☒	☐ ChIP-seq
☒	☐ Flow cytometry
☒	☐ MRI-based neuroimaging

Unique biological materials

Policy information about availability of materials

Obtaining unique materials	Isolate samples are available on request and pending approval by Rigshospitalet Department of Clinical Microbiology, Datatilsynet, and DTU Data Protection Agency.
